# Latent dimensions of financial behaviour derived by principal component and factor analysis

**DOI:** 10.1057/s41264-026-00384-5

**Published:** 2026-08-07

**Authors:** Maryam Sholevar

**Affiliations:** Edinburgh Business School, Heriot-Watt University, Edinburgh, UK

**Keywords:** Financial behaviour, Financial literacy, Financial inclusion, Principal component analysis, Factor analysis, Risk management

## Abstract

Rather than directly measuring the dimensions of financial behaviour, this study constructs latent dimensions from observed indicators through multivariate analysis. These latent constructs are subsequently aligned with conceptually developed dimensions. Dimensionality reduction of the survey data informs the construction of the latent space. Data were collected using a tailored questionnaire administered to a sub-Saharan African sample (470 participants from urban and rural Ethiopia). Principal component analysis (PCA) and factor analysis (FA) were employed to test the framework and examine interrelationships among dimensions. The results provide exploratory support for aspects of the proposed multidimensional framework; however, further validation through confirmatory factor analysis (CFA), replication, or external behavioural outcomes is necessary. Financial inclusion emerges as a relatively distinct, access-related dimension associated with usage, but its status as a prerequisite for broader financial behaviour requires additional investigation. Financial literacy is most strongly linked to usage and knowledge-related indicators, and while its role in risk management remains conceptually plausible, it requires direct empirical testing. The findings highlight the limitations of inclusion-focused metrics and underscore the multidimensional nature of financial literacy. By integrating a novel conceptual framework, original data, and advanced statistical techniques, this study provides a comprehensive approach to understanding financial behaviour and challenges traditional assumptions, thereby establishing a foundation for future research and more effective strategies in financial education and inclusion.

**Supplementary Information:**

The online version contains supplementary material available at https://doi.org/10.1057/s41264-026-00384-5.

## Introduction

Financial literacy has become a central focus in academic research and policy, particularly following the 2008 global financial crisis, which revealed significant deficiencies in the understanding of financial risk among individuals, institutions, and regulators. Subsequent discourse has increasingly emphasised consumers’ financial knowledge, decision-making capacity, and ability to navigate complex financial products. However, this focus has also exposed a persistent limitation: financial literacy is frequently positioned as the primary explanation for financial behaviour, despite the influence of a broader array of cognitive, behavioural, institutional, and contextual factors. In developing and underbanked contexts, financial choices cannot be adequately explained by standard literacy measures that focus solely on interest rates, inflation, or investment knowledge.

This study adopts the perspective that financial behaviour should be conceptualised as a multidimensional construct, rather than as a direct outcome of financial literacy alone. While financial literacy is a significant factor, it constitutes only one element within a broader construct space that includes financial inclusion, usage of financial services, trust in financial institutions, and risk management. These dimensions are strongly influenced by the social and financial context in which individuals operate. For example, access to financial services may facilitate formal participation but does not necessarily result in active or effective usage. Likewise, financial knowledge may enhance decision-making but does not automatically lead to risk-aware behaviour or confident financial planning.

The need for a broader analytical framework is especially pronounced in contexts where financial access, service availability, institutional trust, and informal financial practices vary substantially across populations. Accordingly, this study utilises original survey data collected from 470 participants in urban and rural areas surrounding Jimma, Ethiopia. The Ethiopian context is particularly valuable for examining dimensions of financial behaviour, as individuals’ experiences with formal financial services vary considerably by employment status, gender, location, income source, and level of institutional access. Rather than relying on narrow or externally standardised measures of financial literacy, this study employs a tailored questionnaire that captures multiple observable indicators, including inclusion, literacy, usage, trust, and risk management.

Principal component analysis (PCA) and factor analysis (FA) are employed to determine whether the observed indicators coalesce into coherent latent structures. PCA is utilised to investigate the organisation of variance among the indicators, while FA assesses whether the observed relationships among variables support the existence of interpretable latent dimensions. These methods are not intended to establish causal pathways or predict downstream outcomes such as wealth, savings growth, credit performance, or financial well-being. Rather, they serve as exploratory tools for assessing constructs, evaluating the empirical plausibility of the proposed multidimensional framework, and identifying areas that may require refinement.

Accordingly, this study pursues four interrelated objectives. First, it reconceptualises financial behaviour as a broad latent construct encompassing financial inclusion, financial literacy, usage, trust, and risk management. Second, it develops and implements original survey indicators to operationalise these dimensions within a sub-Saharan African context. Third, it applies PCA and FA to evaluate whether the statistically derived latent dimensions correspond to the conceptually proposed dimensions. Fourth, it identifies which dimensions are empirically distinct, which overlap, and what refinements are necessary for future confirmatory research. The contribution of this paper is exploratory and framework-oriented; it does not seek to establish a causal model of financial behaviour but instead provides a structured foundation for future studies to test the proposed relationships using confirmatory, longitudinal, or behavioural outcome data.

## Literature review and conceptual framework

### Financial literacy: prominence, ambiguity, and contextual limits

Although financial literacy had been discussed since the 1970s, the 2008 global financial crisis brought it to the forefront as a central academic topic and policy concern, exposing widespread gaps in the public’s understanding of financial systems (Goyal and Kumar 2021). The 2008 crisis was closely linked to subprime mortgage losses and securitisation, as well as wider failures in regulation, supervision, institutional risk management, and credit ratings. One possible theory is that poor financial decisions stem from shortsightedness arising from a lack of understanding of financial products. Post-crisis policy discourse increasingly emphasised financial education and individual capability, sometimes alongside critiques that this emphasis may underplay structural causes. In fact, it has become common to highlight the lack of financial literacy among consumers during crises, from the 2008 crash to the COVID-19 pandemic (Knowles and Schifferes 2020; Gerth et al. 2021; Bechlioulis and Karamanis 2023; Setiawan et al. 2024).

As a result, financial literacy became more prominent in post-2008 policy and research agendas and was frequently referenced by major financial institutions and federal bodies in the United States. Prominent studies, such as those by Lusardi and Mitchell (2011), established a foundation for subsequent research by linking financial literacy to wealth accumulation, retirement planning, and overall financial well-being. Cude (2021) explained that Lusardi and Mitchell did not provide a simple definition of financial literacy in their pioneering works. This lack of definitional clarity has prompted critiques, including concerns over the universal applicability of their framework and the appropriateness of their assessment methods. Studies such as those by Klapper et al. (2015) highlight the limited global applicability of standardised measures of financial literacy, noting significant disparities across financial systems, product access, and educational priorities. Atkinson and Messy (2012) argue that the focus on interest rates and investment products overlooks basic financial practices prevalent in developing and underbanked regions. In most countries, individuals’ primary concern is access to basic financial services rather than credit. Even in the United States, 4.5% of individuals are still unbanked (Federal Deposit Insurance Corporation, 2022). In many cases, people use complex financial loans (including unregulated markets) whose overall interest rates can be devastating, and a rudimentary understanding of interest rates might even exacerbate the illusion of affordability.

Therefore, there is a vital need to move away from the established oversimplification of financial literacy. Stella et al. (2020) utilised principal component analysis and proposed a new questionnaire to measure financial literacy across three main dimensions: attitude, skills, and knowledge. This reflects the broader need to treat financial literacy not as a single narrow score, but as a construct whose meaning depends on the financial, institutional, and social context in which it is measured.

### From financial literacy to financial capability and behaviour

A useful theoretical entry point lies in a limitation exposed by linguistics. While financial literacy can be redefined, it remains constrained by the established meaning of literacy itself. Financial literacy is often compared with reading literacy and assumed to be essential for sound financial decisions, just as literacy is required to read a book. Cude (2021) reviewed various definitions of financial literacy in the literature and found that almost all centred on “sound financial decision-making.” However, Huston (2010) criticises this focus, stating that most definitions fail to capture the behavioural, cultural, and contextual aspects critical to real-world applications. Cude (2021) also elaborated on the vagueness of financial literacy definitions in the literature. This ambiguity has led to inconsistencies in how financial literacy is operationalised across studies, hindering the establishment of a robust link to financial behaviours. Huston (2010) suggested that a lack of conceptual clarity in financial literacy studies can hinder the formulation of actionable insights.

The problem arises from oversimplifying this analogy. In using financial services, there is no equivalent of “reading”; instead, it is akin to “writing”. Literacy enables someone to read, but it does not make them a creative writer. Similarly, financial literacy enables an individual to comprehend financial services, but it does not inherently translate into effective financial decision-making. A bank clerk, for example, may be financially literate enough to manage daily transactions but may lack the authority or skill to make strategic financial decisions. Remund (2010) categorised financial literacy into five domains: knowledge, skills, attitudes, behaviour, and financial well-being, stressing that literacy alone is insufficient without practical application.

The key difference is that financial behaviour is often considered a causal effect of financial literacy. However, financial behaviour encompasses financial decisions and actions, while various types of knowledge can serve as the basis for these actions. Xiao and Porto (2017) argued that financial literacy is only one predictor of financial behaviours and does not necessarily translate into optimal financial outcomes. Therefore, it is more reasonable to consider financial behaviour as a general, multidimensional construct in which financial literacy is a contributing cognitive factor alongside behavioural and perceptual elements. Ingale and Paluri (2022) surveyed the relationship between financial literacy and financial behaviour in the literature and highlighted a shift towards a psychological and behavioural perspective. Supporting this trend, Farrell et al. (2016) integrated psychological frameworks, such as bounded rationality and decision-making heuristics, to explain the linkage between financial literacy and behaviour more effectively. Financial inclusion is a contributing factor in financial behaviour, since a financially excluded person may still exhibit certain financial behaviours, albeit measured differently.

Financial decisions are influenced by a complex interplay of psychological, economic, cultural, and social factors (Dervishaj 2021; Ingale and Paluri 2022; Lone et al. 2025). Financial literacy is only one of these contributing factors. Lusardi et al. (2017) reinforce this point, suggesting that while financial literacy supports decision-making, it is moderated by factors such as risk tolerance, social norms, and cognitive biases.

Thus, financial literacy can be compared with writing skills. When testing writing skills (e.g., in a language learner), the goal is to evaluate their ability to construct grammatically correct sentences, not necessarily their creativity. Education focuses on teaching standard structures to ensure clarity and correctness. In real-life writing, however, creativity becomes essential for impactful communication. Estelami and Estelami (2024) noted that financial literacy is not necessarily determined by education and that cognitive styles may play a crucial role. Similarly, financial literacy and risk management are aligned in sequence. Financial literacy represents foundational knowledge that can be formally taught and measured, while risk management encompasses the experiential and adaptive application of that knowledge. Sherraden (2013) demonstrated that financial capability combines knowledge, opportunity, and context to influence behaviour effectively.

The central point is that financial decisions belong more appropriately to risk management than to financial literacy. Financial literacy enables individuals to take calculated rather than blind risks. Xu and Zia (2012) highlighted that financial education should emphasise practical decision-making skills rather than rote learning of financial concepts. For example, even a simple credit card transaction involves risk management, as failure to meet repayment deadlines can trigger a domino effect on an individual’s financial stability and credit history.

The global financial crisis of 2008 serves as a cautionary tale often attributed to a lack of financial literacy. The common narrative suggests that people took on unaffordable risks by taking out subprime mortgages. However, this was as much about risk management as it was about financial literacy. Many individuals took calculated risks that, under normal circumstances, might have been manageable. Agarwal et al. (2014) argue that structural issues, such as predatory lending practices and inadequate regulation, played a far greater role than individual knowledge deficits. Moreover, many financially literate, risk-averse individuals also suffered significant losses by investing in institutions that ultimately failed.

For instance, the cryptocurrency boom provides another illustrative example. Many beneficiaries of the boom were younger individuals, largely due to their exposure to digital platforms and willingness to adopt innovative financial products. Yang et al. (2023) underlined how technological familiarity and risk-taking behaviour could outweigh traditional financial literacy in influencing financial success in such contexts. Instead, different dimensions of financial literacy must be identified and linked to other financial concepts. Hassan et al. (2024) highlighted the impact of various factors that may lead a person to utilise fintech actively. Fernandes et al. (2014) conducted a meta-analysis revealing that financial literacy explains only a small proportion of the variance in financial behaviour, underscoring the need for multidimensional approaches.

Taken together, these arguments align with the financial capability perspective (Sherraden 2013), in which knowledge, opportunity, and context jointly shape behaviour. They motivate an umbrella construct, financial behaviour, within which literacy is one of several contributing dimensions, alongside inclusion, usage, trust, and risk management. The present focus is therefore to place financial literacy and inclusion under the umbrella of financial behaviour to examine the latter. Morgan and Trinh (2019) emphasised the interconnection between financial inclusion and financial behaviours, particularly in developing regions. Therefore, the financial behaviour discussed throughout this paper is linked to financial literacy and inclusion.

### Evidence on the dimensions of financial behaviour

Although the definition of financial inclusion is not well established in the literature, the common perception is that it is a static concept. In general, financial inclusion/exclusion is often equated with being banked or unbanked (Chaia et al. 2009; Liyanage et al., 2024; Ellili 2024). The basic idea is that having a bank account can pave the way for access to financial services. However, studies such as Sarma and Pais (2011) have highlighted the multidimensionality of financial inclusion, with access, usability, and service quality playing critical roles beyond mere access. It is, of course, a subject of debate whether being banked is sufficient and/or necessary for financial inclusion. For example, Beck et al. (2009) note that having a bank account alone does not necessarily translate into effective financial inclusion if services are inaccessible or underutilised.

The starting point is financial inclusion, which serves as a prerequisite for financial actions but does not guarantee them. Financial inclusion is also inherently static; for example, an individual with a dormant bank account is considered financially included yet may not actively use financial services. This static nature of financial inclusion is critiqued by Sarma and Pais (2011), who argued that access alone is insufficient and that the quality and frequency of engagement are vital indicators of true financial inclusion. While thresholds for active inclusion can be adjusted (e.g., focusing on active accounts), activity may still be limited to basic or automated transactions. At the basic access stage, financial literacy may be less decisive than structural barriers, affordability, documentation, trust, and service availability. Demirgüç-Kunt et al. (2018) suggest that trust and institutional inefficiencies are among the most significant barriers to financial inclusion, particularly in developing regions. Evidence from sub-Saharan Africa reinforces this contextual view: even within the region’s growing middle class, consumers are heterogeneous in their financial realities, and the availability, accessibility, and affordability of services (together with trust in providers) rather than access alone drive the selection and consumption of financial services (Chikweche et al. 2024).

In this context, financial inclusion is better understood as a societal concept rather than a purely economic one, with greater emphasis on inclusion than on finance. Financially, the critical dimension is usage, which assesses the benefits individuals derive from financial services relative to their potential. Usage highlights active engagement with financial services, including transactions, savings, and credit use. Beck et al. (2009) reported that active usage is a key determinant of the economic impact of financial inclusion, linking it to improved financial health and economic stability. Here, financial literacy becomes crucial, as it facilitates informed decisions and active participation. However, financial literacy is a multidimensional construct that varies across different levels and contexts.

The boundaries of financial literacy are largely dictated by the availability of services (Sholevar and Harris 2020). Huston (2010) argues that financial literacy should be evaluated relative to the financial ecosystem in which individuals are exposed, as benchmarking against unavailable services introduces significant biases. For instance, assessing financial literacy based on familiarity with investment products in a population where such products are inaccessible is neither practical nor meaningful.

Financial literacy is a prerequisite for effective risk management, but it does not guarantee successful risk management. Fernandes et al. (2014) highlight that while financial literacy supports risk awareness, other factors, such as emotional intelligence, social influences, and economic conditions, also play pivotal roles in risk management. Risk management, in this sense, involves applying financial literacy to evaluate and mitigate potential risks. Financial literacy is an efficient tool for managing risks, but it must be complemented by behavioural competencies, such as the ability to weigh long-term consequences against short-term benefits (Siriopoulos 2021). This is further supported by Remund (2010), suggesting that financial literacy must extend beyond knowledge to include the ability to apply concepts in complex, real-world scenarios.

By framing financial inclusion, usage, and risk management as interconnected yet distinct dimensions, this study builds a comprehensive understanding of financial behaviour. Collins et al. (2009) argued that a dynamic interplay of structural, educational, and behavioural factors shapes financial behaviours.

### Multivariate analysis of financial literacy and behaviour

Multivariate analysis is a first step in understanding high-dimensional data (Backhaus et al. 2021), and financial behaviour falls into this category. Although multivariate analysis has been used in the context of financial literacy, it is not a common research strategy, as one may expect. Part of this reluctance may stem from the prevalence of simplified measures of financial literacy that fail to capture its multidimensional nature. When inspecting financial literacy with five closely related questions, there is little room for multidimensional manoeuvres. Quite interestingly, several prominent multivariate studies of financial literacy have focused on developing-country contexts (Murendo and Mutsonziwa 2017; Nanziri and Leibbrandt 2018; Ahmad et al. 2020), where standard short financial-literacy question sets may be insufficient for capturing context-specific multidimensional behaviour, and the research had to extend the questionnaires to get meaningful responses. This reflects an inherent limitation in globally standardised surveys, which often overlook contextual and cultural variations (Morgan and Trinh 2019).

Principal component analysis (PCA) and factor analysis (FA) are leading techniques for unravelling complex relationships in high-dimensional datasets. They can be used to validate conceptual frameworks statistically, thereby bridging the gap between theoretical constructs and empirical evidence (Henson and Roberts 2006). PCA has been successfully employed to construct a composite financial literacy score (Murendo and Mutsonziwa 2017; Nanziri and Leibbrandt 2018).

The application of FA extends beyond dimensionality reduction, offering a framework for deciphering underlying structures and their impact on observed variables. For instance, Farrell et al. (2016) used FA to investigate the interplay of psychological and socio-economic variables in shaping financial behaviour. This illustrates its versatility in revealing hidden patterns that inform targeted interventions and policy design. While both PCA and FA are dimensionality reduction techniques, they were chosen for distinct and complementary purposes in this study. PCA indicates the relative contribution of variables or components to explained variance, while FA provides exploratory evidence about whether the observed indicators form coherent latent structures.

The methodological implication of this literature is that a multidimensional construct such as financial behaviour should not be reduced to a single literacy score without first examining whether its indicators form coherent latent structures. The present study therefore uses a theory-informed exploratory design: conceptually defined dimensions are operationalised through survey indicators and then assessed using PCA and FA.

### Conceptual framework, research objectives, and research questions

The recent literature on financial inclusion and literacy emphasises their interdependence, with causality often discussed to clarify the direction of their relationship. However, the present conceptual model defines financial inclusion and literacy as dimensions of financial behaviour, which may interact but do not necessarily determine one another. Atkinson and Messy (2012) argued that financial literacy should not be oversimplified as a linear determinant of financial outcomes, but rather understood as part of a broader framework of financial capability.

**Fig. 1:**
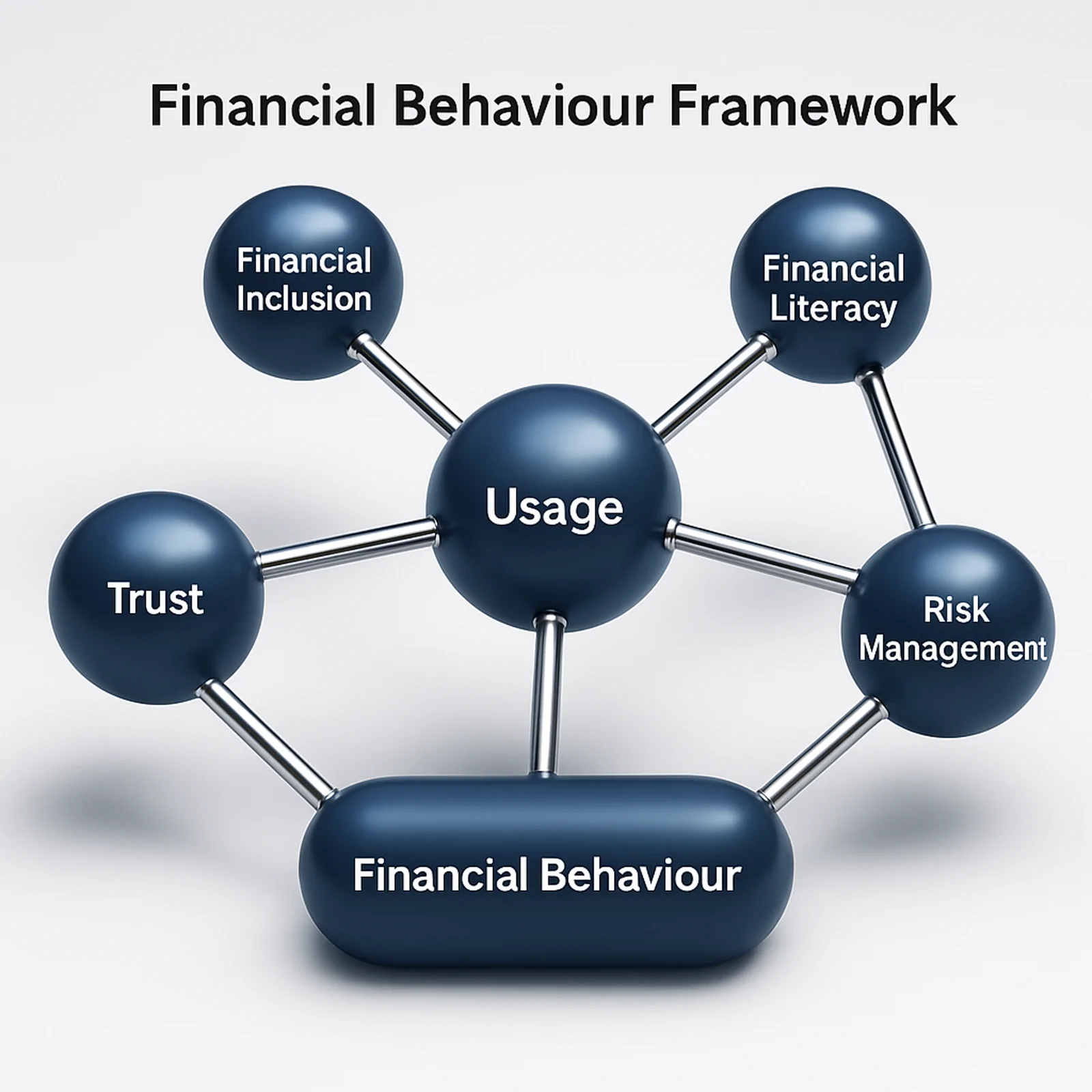
Illustration of the dimensions of financial behaviour

Accordingly, in the present study, financial behaviour is defined as a multidimensional construct encompassing not only observable actions but also the knowledge, attitudes, and contextual factors that enable and shape them. While some researchers, such as Xiao (2008), define financial behaviour more narrowly as observable actions, this framework adopts a broader perspective. Financial behaviour is conceptualised as an ecosystem in which cognitive dimensions (e.g., financial literacy), perceptual dimensions (e.g., trust), and contextual prerequisites (e.g., financial inclusion) interact to produce behavioural outcomes (e.g., usage). Figure 1 illustrates the proposed relationships among these components, which together constitute the financial behaviour construct investigated in this study. It should be stressed that the questionnaire does not include direct behavioural metrics (e.g., saving rates and loan defaults). The indicators capture access, knowledge and attitudes that theory links to behaviour.

The framework builds on the insights reviewed above by treating financial inclusion as a static yet foundational prerequisite for financial behaviour. This contrasts with studies that focus solely on access, as it incorporates a broader perspective on the structural barriers and enablers that influence financial inclusion.

Building on the literature gaps mentioned above, this study pursues four linked but deliberately bounded objectives. First, it re-specifies financial behaviour as a latent construct encompassing financial inclusion, usage, literacy, trust, and risk management, rather than equating it solely with a causal effect of literacy. Second, it develops original survey items, administered to an urban/rural Ethiopian sample, that operationalise each conceptual dimension through observable indicators. Third, it uses PCA and FA to assess whether the latent dimensions in the data align with the conceptual dimensions of financial behaviour. Fourth, the present paper stops at construct validation; it does not model downstream outcomes (e.g., financial well-being) or causal paths among dimensions. Those empirical links are left explicitly for a follow-up study.

Therefore, this study is designed to address these identified gaps directly. It proposes and empirically tests a novel conceptual framework that recalibrates the relationship among key financial concepts, positioning financial behaviour as the overarching construct rather than a consequence of financial literacy. This directly challenges the definitional ambiguity highlighted by Cude (2021) and others. To overcome the limitations of US-centric models and globally standardised surveys, this research utilises original data collected through a tailored survey administered to a sub-Saharan African population. This specific demographic offers a rich and diverse set of financial experiences that are essential for developing a more universally applicable understanding of financial behaviour. The findings provide exploratory support for partial alignment between the conceptual framework and the observed indicator structure, offering a more robust methodology for future research in this field. The analyses are therefore validation-oriented rather than predictive.

Based on this framework, and consistent with the exploratory, framework-generating design, the analysis is guided by three research questions:

RQ1: Do the observed survey indicators cohere into latent dimensions that align with the five conceptual dimensions of financial behaviour (financial inclusion, financial literacy, usage, trust, and risk management)?

RQ2: How are the conceptual dimensions and the indicators underlying them interrelated?

RQ3: Which dimensions are empirically distinct, which overlap, and what refinements to the conceptual framework do the data suggest?

Because the study derives structure from the data rather than testing a pre-specified causal model, directional hypotheses are not imposed at this stage. Instead, the validated structure is used to generate testable hypotheses for future confirmatory research.

## Methodology

### Target population

Ethiopia was chosen as an appropriate target context because of its significant diversity in access and privilege across domains ranging from the social to the financial. For instance, because public education is widely available in developed countries, it is challenging to assess the impact of literacy and financial literacy. The target population was selected from Jimma (a major city in Ethiopia, located in the centre of a geopolitically and economically important region) and its surroundings, including both rural and urban populations. The surveys were conducted in collaboration with a specialised and specifically trained group of researchers from the College of Business at Jimma University. The statistical data derived from the responses are presented in Table S1.

### Survey design and data collection

The survey was a relatively comprehensive questionnaire administered through structured interviews lasting 45–60 min. The interviews were conducted orally in the participants’ local languages by the partner university in Ethiopia. Interviewers collected responses but did not process the data, reducing interviewer-induced bias. The surveys were designed and prepared in the UK, while the partner university conducted the interviews under a contractual arrangement. Only those questions with score-based answers were selected for the statistical analysis.

### Sampling

Due to the heterogeneous distribution of the target population, cluster sampling was employed for Jimma town and district, stratified by employment status and gender, followed by quota-based convenience sampling within the strata. The intention was to achieve proportional ratios of urban versus district respondents and female versus male respondents, although fully proportional sampling could not be achieved. For instance, there is a huge gender gap in the Ethiopian workforce, particularly at the level where financial behaviours are tangible. In the first step, the target population (Jimma town and district) is selected using a cluster sampling approach. The target population was then stratified by employment status (employed, micro-enterprise, and small-enterprise groups) and by gender (male and female). In the final stage, convenience sampling is used to determine the number of respondents in each group. In a developed country, the likelihood of comparable education, income, and social status among men and women is much higher than in a developing country. Hence, an unstratified random sampling method in a developing-country context can be misleading, especially when the research focuses on the depth of inclusion. Data from 470 participants were used in the present statistical analysis. Because key subgroups were unevenly distributed, stratification and quota controls were used to ensure representation across gender, employment status, and urban/rural location. Convenience sampling was then used within strata.

### Multivariate statistical analysis

The analytical strategy maps directly onto the research questions: correlation analysis addresses RQ2 at both the dimension and indicator levels; PCA and FA address RQ1 by testing whether statistically derived dimensions align with the conceptual ones; and the comparison of alternative component and factor solutions addresses RQ3. Table 1 summarises this mapping.

**Table 1:** Mapping of research questions to analyses and outputs

Research question	Analysis	Main outputs
RQ1: Alignment of latent and conceptual dimensions	PCA; FA (two- to seven-factor solutions)	Figs. 3, 4, 5 and 6; Tables S3–S5
RQ2: Interrelationships among dimensions and indicators	Correlation analysis	Figure 2; Table S2
RQ3: Distinctiveness, overlap, and framework refinement	Comparison of PCA and FA solutions	Figs. 3, 4, 5 and 6

Note. PCA = principal component analysis; FA = factor analysis. RQ = research question

All statistical analyses were conducted in R, and the direct outputs (both data and visualisations) were used without further editing. Correlation matrices were obtained using the *corrplot* package. *FactoMineR* and *psych* packages were used for PCA and FA calculations.

Before conducting FA, the dataset’s suitability was formally assessed using two standard statistical tests. Bartlett’s test of sphericity was used to test the null hypothesis that the variables in the correlation matrix are uncorrelated (i.e., the matrix is an identity matrix). A statistically significant result (*p* < 0.05) is required to reject this null hypothesis, indicating that the data are likely suitable for factor analysis. Additionally, the Kaiser-Meyer-Olkin (KMO) measure of sampling adequacy was employed. KMO assesses the proportion of variance among variables that might be common variance. It provides a measure for each variable and an overall score, with values closer to 1.0 indicating ideal performance. A value of 0.60 is often considered the minimum acceptable threshold for proceeding with FA. As will be discussed later, the collected data satisfy these requirements.

For FA, GeominQ was adopted after comparing it with several alternatives. Because the latent dimensions are expected to correlate, an oblique rotation was required. Promax, which starts from a Varimax solution and then allows factor correlations, is computationally inexpensive. However, when items exhibit genuine cross-loadings, it often leaves sizable “secondary” loadings. GeominQ, by contrast, explicitly penalises those small cross-loadings, yielding a clearer near-simple structure while still permitting factor correlations. Notably, the GeominQ solution closely matched the pattern obtained with the CF-cluster rotation criterion, consistent with our goal of isolating conceptually coherent indicator clusters.

### Terminology and measurement

Before detailing the dimensions, it is crucial to distinguish between the latent constructs this study investigates and the observed variables used to measure them. A latent construct (e.g., financial literacy) is an abstract, unobservable concept. An observed variable is a concrete, measurable indicator derived directly from the survey questionnaire (e.g., the score on a specific numeracy question). In this study, a set of observed variables is used to statistically infer and validate the existence and structure of the five proposed latent constructs: financial inclusion, financial literacy, usage, trust, and risk management. The subcategories detailed below (e.g., numeracy, access) represent the specific facets or factors that comprise these broader constructs.

Throughout this paper, a three-tier vocabulary is used: item (a single-scored questionnaire question), indicator (a short item bundle that operationalises a facet, e.g., numeracy), and latent construct (one of the five theoretical dimensions). PCA and FA reduce a set of indicators into latent constructs. These techniques are not merely statistical tools; they serve as bridges between theoretical constructs and empirical data, enabling researchers to validate and refine conceptual frameworks (Henson and Roberts 2006). To understand the meaning of the resulting dimensions, a clear grasp of the underlying dimensions is needed. The dataset’s starting dimensions are the scored questionnaire items. The primary step is to define what factor each question represents. This process, while rooted in statistical analysis, often involves subjective judgement and theoretical interpretation. For instance, when examining the underlying factors of financial behaviour, a single question may represent several factors to varying degrees. Fabrigar et al. (1999) argue that the subjective nature of factor identification can be mitigated by combining statistical rigour with theoretical grounding.

Table 2 lists questionnaire items, the indicator each loads on, and the target latent constructs.

Following this distinction, five key latent constructs (hereafter referred to as dimensions) of financial behaviour were identified for this research: financial inclusion, financial literacy usage, trust, and risk management. This set of dimensions is not exhaustive; additional dimensions may be considered.

Although these dimensions have been extensively studied in the literature, there is no consensus about their subcategories. The questionnaire used in this study was designed to align with standard, widely used questions, but researchers often classify such questions differently within their specific frameworks. This study aimed to use terminology that is not only sensible but also commonly used in the literature, ensuring clarity and relevance in defining the underlying categories of the dimensions.

**Financial inclusion** is measured by two categories: means and access, representing the reason for inclusion and the degree of success, respectively. In the target population of the present study, any source of income or capital is considered a means; however, other means, such as credit, could also be included. Demirgüç-Kunt et al. (2018) highlight that financial inclusion extends beyond access to encompass affordability and service quality. This broader interpretation underscores the importance of tailoring inclusion metrics to contextual realities.

**Financial literacy** is divided into four categories: numeracy, awareness of available services, basic knowledge, and extended knowledge. Numeracy is the most commonly examined concept in the literature, reflecting the ability to perform basic financial calculations. Awareness focuses on knowledge of accessible financial products and services, while the two levels of knowledge, basic and extended, capture general familiarity with financial concepts and a deeper understanding of specific financial instruments, respectively. Huston (2010) treated financial knowledge as a central component of financial literacy, but it must be contextualised to the financial environment of the target population.

**Usage** is categorised into two primary subcategories: transaction and saving. These represent active engagement with financial services. More advanced uses, such as accounting and planning, involve maintaining detailed transaction records and formulating long-term financial strategies. These advanced forms of usage may also overlap with financial inclusion, reflecting the dynamic interplay between these dimensions. Beck et al. (2009) note that financial service usage is a key determinant of the effectiveness of financial inclusion, with savings often serving as a critical indicator of financial stability. The relationship between usage and financial behaviour concerns what is used versus how it is used.

**Trust**, as a dimension, is inherently complex. It encompasses several subcategories, each reflecting different aspects of confidence in financial systems. Compared to other dimensions, trust is more subjective, as it is primarily derived from opinion-based responses rather than quantifiable data (Sholevar and Bachmann 2025). Guiso et al. (2008) argue that trust is a cornerstone of financial behaviour, particularly in environments where formal institutions lack transparency or accountability. The terminology used in this study for trust is adapted from the existing literature to align with commonly accepted definitions.

**Risk management** is the final dimension, though its scope was limited in this study because the target population lacked high-risk financial products. The purpose was to assess openness to risky options beyond standard financial practices. Lusardi et al. (2017) emphasised that risk management is a crucial component of financial capability, encompassing both risk identification and the development of strategies to mitigate them. This dimension highlights the practical application of financial literacy in real-world decision-making (Ananda et al. 2024).

By categorising financial behaviour into these dimensions and their subcategories, this study establishes a comprehensive framework for investigating the interrelationships among financial behaviours. Such an approach contributes to the ongoing discourse on the multidimensional nature of financial capability, as advocated by Sherraden (2013).

### Analysis and findings

The findings are reported in three steps, each tied to the research questions: correlation analysis (RQ2), principal component analysis (RQ1 and RQ3), and factor analysis (RQ1 and RQ3). Table 1 summarises this mapping. All figures report unedited outputs of the analyses, and the underlying numerical results are provided in Supplementary Tables S2-S5.

### Correlations

**Fig. 2:**
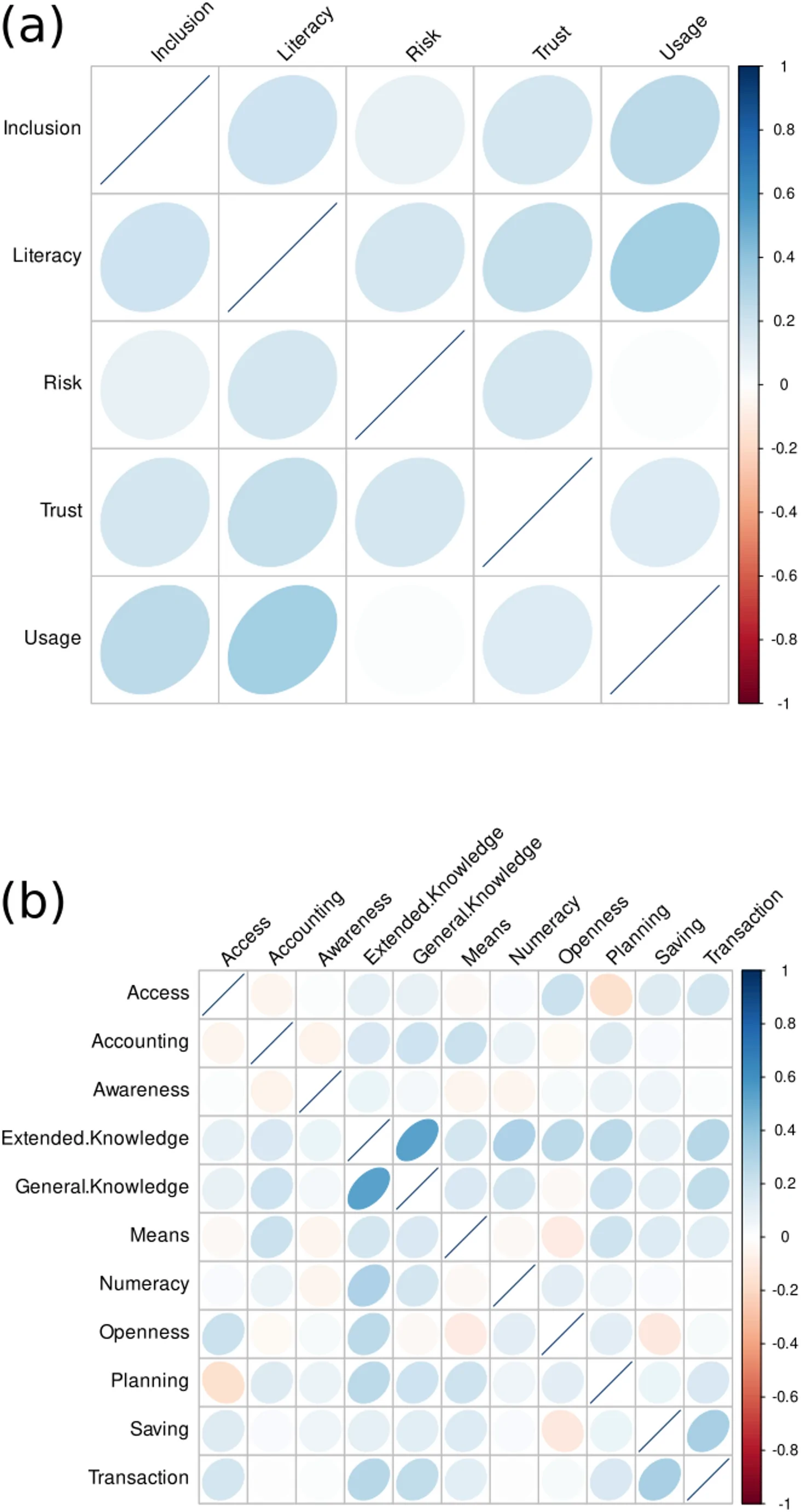
Correlation matrices of (**a**) dimensions of financial behaviour, and (**b**) underlying factors of the dimensions of financial behaviour

The primary approach to understanding the anatomy of multidimensional data is to examine the correlation matrix, which helps pinpoint the prominent relationships among the dimensions (Fig. 2a and b). The strongest correlation is observed between financial literacy and usage, as expected and widely supported in the literature (see Table S2 for numerical data). Specifically, the correlation coefficient of 0.333 between literacy and usage indicates a moderate positive relationship, suggesting that individuals with higher financial literacy are more likely to actively use financial services. Lusardi and Mitchell (2014) emphasised the role of financial education in enhancing financial engagement. Similarly, the correlation between financial inclusion and usage is also expected. With a correlation coefficient of 0.252, inclusion and usage demonstrate that access to financial services increases the likelihood of their use, reinforcing the notion that financial inclusion is a critical foundation for active financial behaviour. The relationship between trust and financial literacy may be important for marketing purposes. The positive correlation of 0.234 between literacy and trust suggests that greater financial knowledge fosters greater confidence in financial institutions, a dynamic that marketers can strategically leverage to build customer loyalty and trust-based relationships. The near-zero correlation between usage and risk is particularly interesting. This finding challenges the assumption that increased usage of financial services leads to higher risk-taking, suggesting that active engagement does not inherently drive individuals towards riskier financial products. Openness to new financial services might be interpreted as a step towards more complex financial services. However, the data show that greater use does not push people towards riskier products.

At the indicator level, interesting correlations between the underlying factors can be observed (Fig. 2b). The strongest correlation is between *general* and *extended knowledge*, with a coefficient of 0.531, suggesting the progressive nature of financial literacy. This high correlation underscores the continuum of financial literacy in which general knowledge serves as a foundation for more specialised, extended knowledge. Furthermore, both general and extended knowledge show correlations with almost all other factors. General knowledge shows mostly weak positive correlations with access (0.092), means (0.155), awareness (0.044), numeracy (0.170), and transaction (0.242), indicating that foundational financial understanding is linked to various aspects of financial behaviour. *Extended knowledge*, with higher correlations across multiple dimensions, reinforces the idea that deeper financial understanding enhances comprehensive financial engagement.

*Access* and *numeracy* are often considered the main factors of financial inclusion and financial literacy, respectively. However, there is no meaningful correlation between these two factors, as indicated by the correlation coefficient of 0.028. The lack of correlation between *access* to financial services and *numeracy* suggests that access to financial services does not inherently improve an individual’s numeracy, and vice versa. This separation highlights the need for targeted financial education programmes that specifically address numerical competencies regardless of access levels. At the factor level, access shows generally weak-to-modest associations, including with openness (0.213), transaction (0.170), planning (-0.164), saving (0.130), extended knowledge (0.106), and general knowledge (0.092). Specifically, the correlation coefficients between *access* and *risk* (0.096), *trust* (0.184), and *saving* (0.130) are relatively low, indicating that financial inclusion alone does not strongly influence these dimensions of financial behaviour. In other words, financial inclusion/access is most clearly associated with usage-related indicators, while its associations with other dimensions are weak to modest and should not be interpreted causally. Similarly, *numeracy* shows no strong correlation with any factor other than knowledge (both general and extended). This finding highlights the complexity of financial behaviour, in which access and basic numerical skills alone are insufficient to drive comprehensive financial engagement and decision-making.

Both planning and accounting have negative correlations with access, suggesting that access alone is not positively associated with these aspects of financial behaviour. Specifically, *planning* shows a negative correlation of -0.164 with *access*, and *accounting* has a negligible negative correlation of -0.058 with *access*. This suggests that merely having access to financial services does not, in itself, enhance an individual’s ability to plan or manage their finances effectively. It should be noted that planning and accounting do not encompass “the sound financial decision” in the definition of financial literacy; rather, they encompass sensible financial practices. This distinction implies that while individuals may engage in routine financial activities such as budgeting or record-keeping, these practices do not necessarily translate into more informed or strategic financial decisions without comprehensive financial literacy.

### Principal component analysis

PCA is a widely used technique in data analysis and machine learning to reduce the dimensionality of a dataset while preserving most of its important information (Greenacre et al. 2022; Janićijević et al. 2022). The core idea behind PCA is to transform the dataset’s original features into a new set of uncorrelated variables, known as principal components. These principal components are ordered so that the first component captures the maximum variance in the data, followed by the second component capturing the maximum remaining variance, and so on. By retaining only a subset of the principal components that explain most of the variance in the data, PCA effectively reduces the dataset’s dimensionality while minimising information loss. The dimensionality reduction simplifies the dataset, making it easier to visualise, analyse, and model. PCA achieves dimensionality reduction by projecting the data onto a lower-dimensional subspace spanned by the principal components, thereby retaining the most important aspects of the original data while discarding redundant information captured by the less significant principal components.

**Fig. 3:**
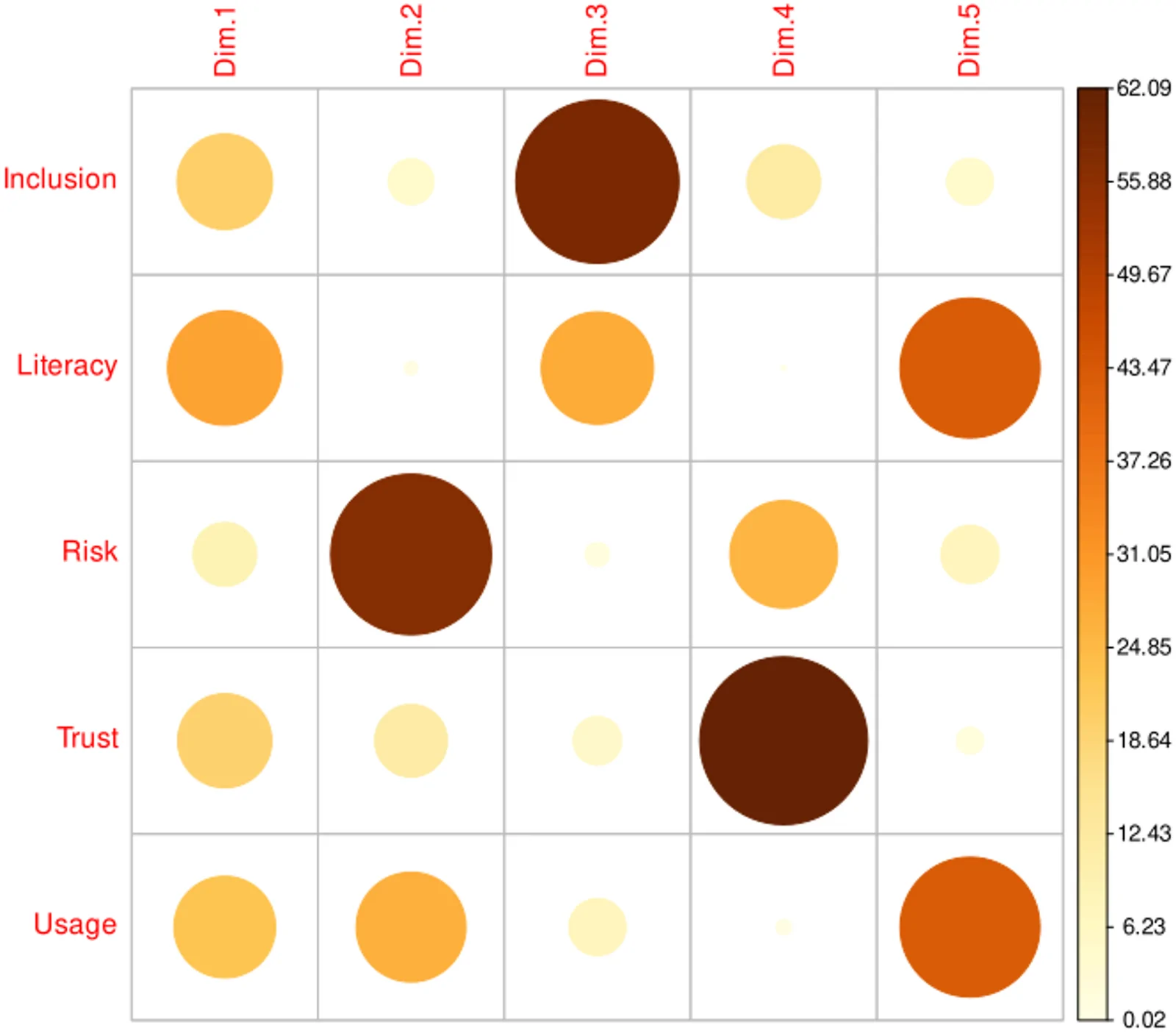
The variance contributions of each conceptual dimension of financial behaviour to the five dimensions derived statistically by principal component analysis

Here, the latent constructs (dimensions) are conceptually defined, and the analysis examines how well the observed variables group together to support this conceptual structure. This approach allows the study to evaluate whether the theoretical framework aligns with the empirical data, thereby reinforcing or challenging the proposed conceptual dimensions. Figure 3 shows the variance contributions of the five conceptual dimensions to the same number of statistically derived dimensions. The eigenvalues indicate the amount of variance each principal component captures. Because PCA was applied to five pre-aggregated dimensions, five PCs necessarily explain 100% of the variance. Therefore, the component loadings should be interpreted rather than the cumulative explained variance. *Inclusion*, *trust*, and *risk* contribute almost independently to their respective dimensions, while *literacy* and *usage* contribute equally to the last dimension. Specifically, the first principal component explains 35.07% of the variance (Table S3), primarily driven by *literacy* and *usage*, suggesting that these two dimensions are highly interrelated and collectively represent a significant portion of the variance in financial behaviour. All five conceptual dimensions contribute to the first latent dimension, which accounts for the largest variance, with *literacy* and *usage* being the two main contributors. This dominance of *literacy* and *usage* in the first principal component underscores their critical role in shaping overall financial behaviour and resembles the centrality of financial knowledge and active engagement (Sherraden 2013). Latent dimensions 1 and 5 suggest a strong connection between *literacy* and *usage*, consistent with the correlation analysis reported in the previous section. This convergence reinforces the notion that *financial literacy* not only enhances *usage* but also interacts synergistically with other dimensions to influence comprehensive financial behaviour.

It is plausible to take a step further and repack the dimensions based on the underlying factor. It should be noted that dimensions should not be derived from assumed factors, as not all factors within a dimension are expected to be aligned. They may present different features, which is why they are considered in the first place. However, this can provide insight into the overall linkage among different factors, in addition to the conceptual classification.

**Fig. 4:**
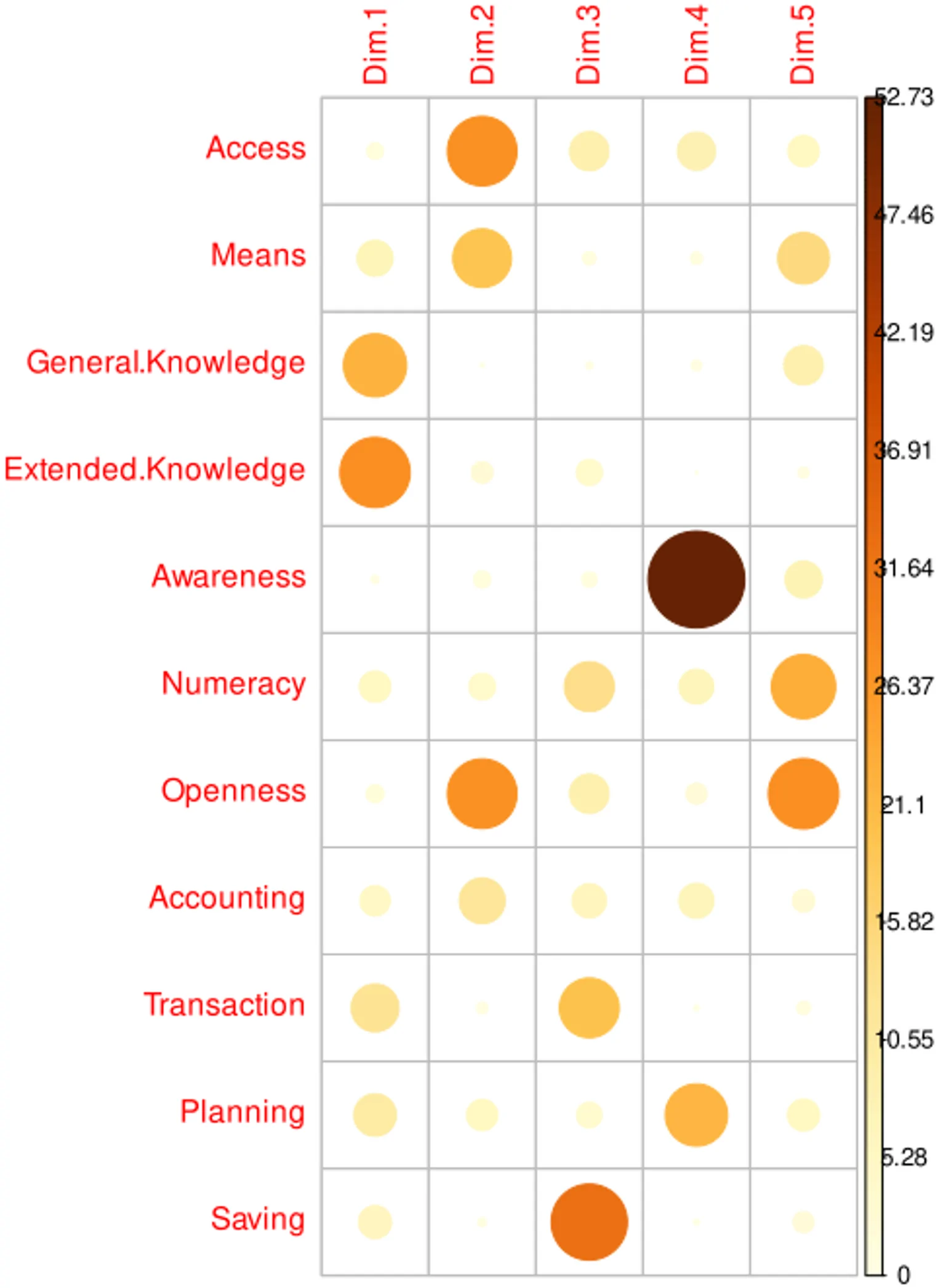
The variance contributions of each factor of the dimensions of financial behaviour to the five dimensions derived statistically by principal component analysis

*General* and *extended knowledge*, the main contributors to the financial literacy dimension, built the first latent dimension (Fig. 4). The second dimension comprises *access* and *means*, both of which are factors in financial inclusion. However, *openness* makes a significant contribution, serving as the sole factor in *risk management*. The third statistical dimension covers *transaction* and *saving*, the main factors of the *usage* dimension. The fourth dimension is primarily influenced by *awareness*, a key factor in financial literacy. Finally, the last dimension primarily encompasses *numeracy* and *openness*, which are key components of financial literacy and risk management. While these results indicate that the overall classification of the factors into the given conceptual dimensions is sensible, there is strong evidence for the complexity of financial literacy as its factors are spread across three latent dimensions. A similar argument can be made for the factors of the *usage* dimension. Nonetheless, there are still dimensional correlations between the factors, such as *general* and *extended knowledge* (in agreement with the previous analysis). This suggests that the underlying factors can be categorised into subcategories when defining the conceptual dimensions.

With respect to RQ1, the PCA results indicate that the conceptual classification of indicators into dimensions is broadly sensible. At the same time, the dispersion of literacy-related factors across several components speaks to RQ3, signalling that financial literacy is not a unitary construct.

### Factor analysis

FA is a statistical technique used to reduce the dimensionality of a dataset by identifying underlying latent variables, or factors, that explain the correlations among observed variables (Shrestha 2021; Allee et al. 2022). Unlike PCA, which focuses on capturing variance in the data, FA aims to model the relationships between observed variables and latent factors. FA assumes that observed variables are linear combinations of unobserved factors plus random error terms. By extracting these latent factors, FA enables the representation of the original dataset using fewer factors, thereby reducing dimensionality while preserving the data’s essential structure. The key difference between PCA and FA lies in their underlying assumptions and goals. PCA is based on the concept of variance maximisation and orthogonal transformations, whereas FA is rooted in factor modelling and correlation structures.

Additionally, PCA produces uncorrelated, orthogonal principal components, whereas FA can estimate correlated factors when an oblique rotation is used. Furthermore, PCA is more suitable for data compression and dimensionality reduction when the relationships between variables are purely linear, and the goal is to capture as much variance as possible. In contrast, FA is often used to investigate latent covariance structures underlying observed variables and to interpret the relationships between variables and latent factors.

Before conducting FA, the suitability of the data were assessed based on the criteria outlined in the methodology. Bartlett’s test of sphericity was highly significant (*χ*2(10) = 158.69, p < 0.001), strongly rejecting the null hypothesis and indicating that sufficient intercorrelations among the dimensions warrant factor analysis. The Kaiser-Meyer-Olkin (KMO) measure yielded an overall Measure of Sampling Adequacy (MSA) of 0.64. While this value is considered middling by common heuristics, it is above the minimum acceptable threshold of 0.60. The MSA values for the individual dimensions were also acceptable: *inclusion* (0.69), *literacy* (0.63), *risk* (0.60), *trust* (0.69), and *usage* (0.60). Overall, these tests confirmed that the dataset, while not exhibiting an exceptionally strong factor structure, is suitable for FA.

**Fig. 5:**
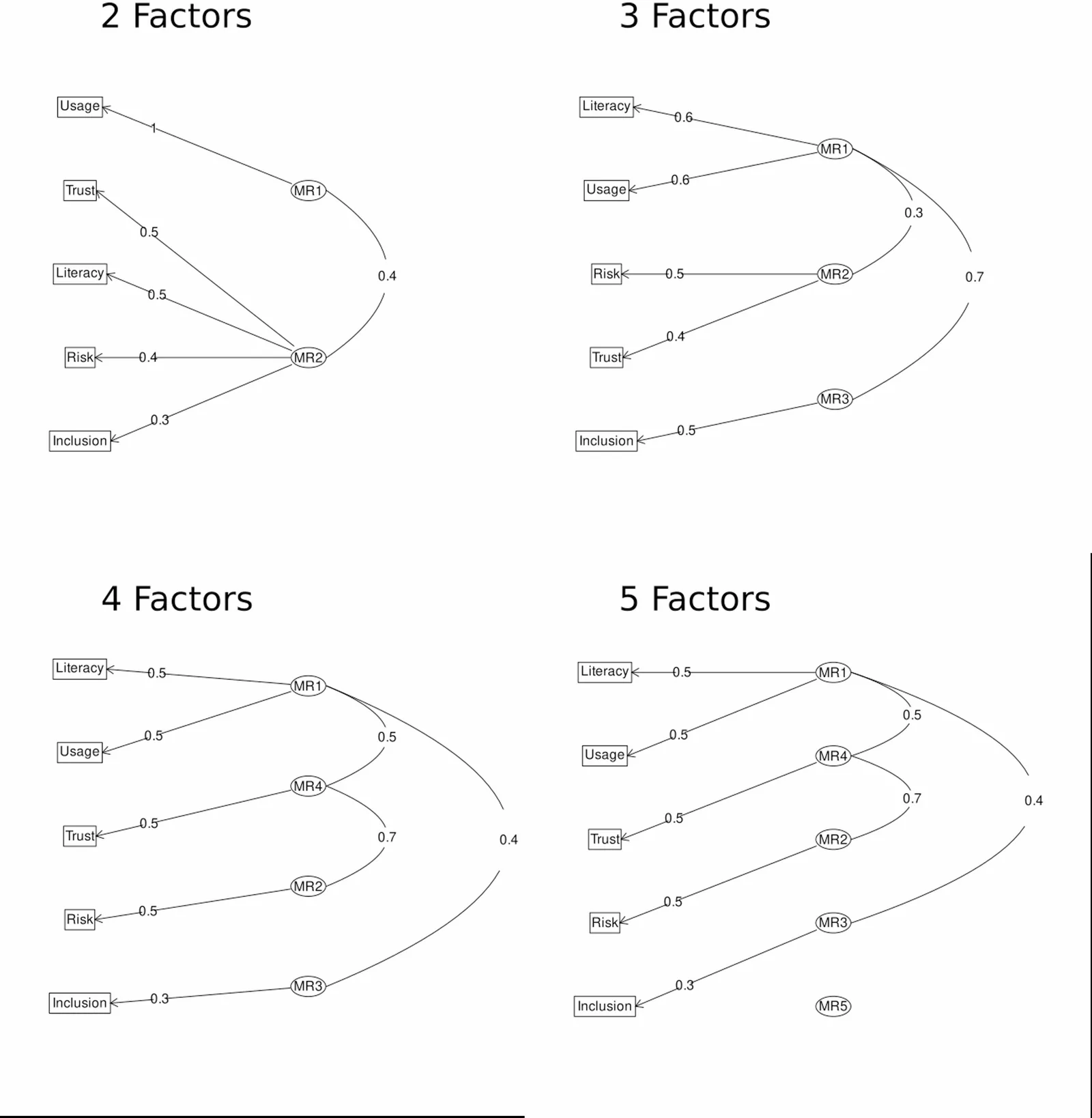
The relationships between the conceptual dimensions of financial behaviour and the 2-, 3-, 4-, or 5-dimensional models derived statistically using factor analysis

FA applied to the five conceptual dimensions (Fig. 5) suggests a dimensionality similar to that observed in PCA. Here, *literacy* and *usage* build the first dimension. The fifth dimension largely duplicates the four-dimensional FA solution and is therefore not interpreted. Reducing the FA solution to three dimensions would require a new conceptual merger of trust and risk. However, FA with two dimensions does not yield a clear separation between *literacy* and *usage*. Notably, literacy, along with inclusion, trust, and risk, contributes to a unified dimension. The scree plot of the dimensions, of course, suggests that three dimensions are the most statistically meaningful solution.

The analysis aimed to identify how well the observed variables load onto these factors, providing insights into the dimensionality and structure of financial behaviour.

FA with two factors yielded a usage-dominated first factor and a trust/risk-oriented second factor; literacy loaded moderately on the first factor rather than forming a clearly separate factor from usage. This two-factor solution does not clearly separate literacy and usage; rather, it posits a broad engagement factor anchored in usage, with literacy contributing only moderately to that factor. The Tucker-Lewis Index (TLI) is saturated at 1, and the likelihood chi-square of 0.47 suggests that the 2-factor model is an excellent fit for the data. A Root Mean Square Error of Approximation (RMSEA) of 0 and a Root Mean Square of the Residuals (RMSR) of 0.01 suggest that little remains to be accounted for by a new factor. *Usage* (0.92) is the unambiguous anchor of Factor 1, and *risk* (0.36) and *trust* (0.38) are the primary drivers of Factor 2. Although the two-factor solution provides an adequate statistical fit, additional exploratory solutions were examined to assess whether theoretically meaningful substructures emerge.

In the three-factor solution, risk loads most strongly on MR2, while trust loads more strongly on MR1; therefore, the result suggests only a partial trust-risk association, not a clear merged factor. The solution is statistically unstable, as indicated by negative degrees of freedom (df), and the third factor does not significantly contribute to the formation of a new loading regime. There is indeed a known Heywood case in which the factors are overparameterised. However, within the present approach, it remains justifiable to test how the dimensions are distributed across additional factors.

The four-factor solution does not provide a clean refinement: MR1 remains shared by literacy usage, trust, and inclusion, while MR2 is mainly associated with risk and negatively associated with usage; the additional factors are weak. However, the fourth factor contributed minimally, with numeracy and openness loading weakly, suggesting that these factors may not form a cohesive dimension within this model. The model’s fit remained consistent, but the complexity increased without significant gains in explanatory power.

FA with five factors did not substantially improve the model. Because solutions with three or more factors have negative degrees of freedom, the three-factor and higher-factor models should be treated as exploratory diagnostic decompositions rather than as evidence of stable additional dimensions. This test, despite going beyond the essential purpose of FA, highlighted the primary contributions of *literacy*,* usage*, *trust*, *risk*, and *inclusion*, though overlapping loadings suggest that some dimensions are not entirely independent.inancial literacy usage.

**Fig. 6:**
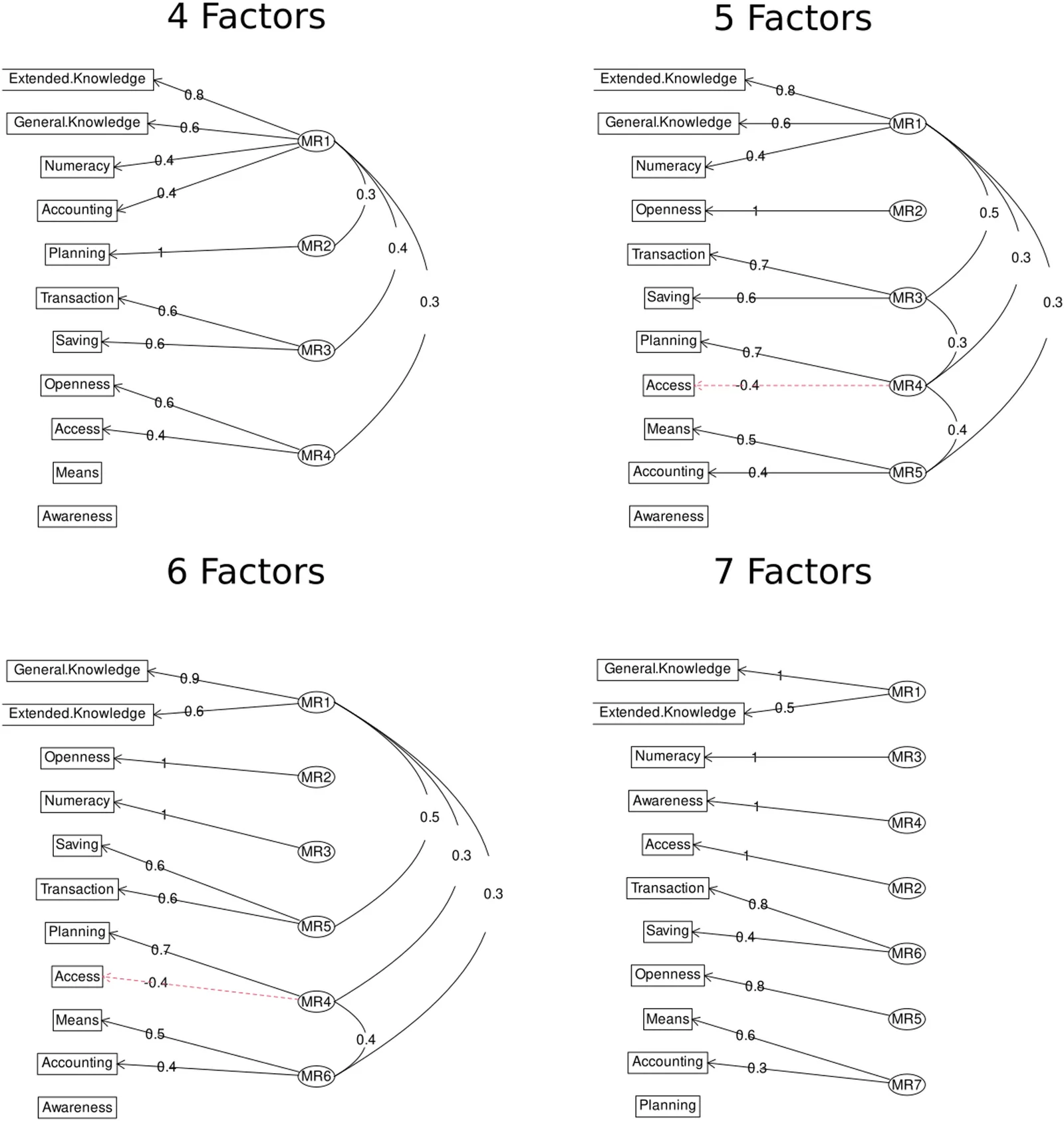
The relationship between the conceptual dimensions of financial behaviour and the 4-, 5-, 6-, or 7-dimensional factors derived statistically using factor analysis

Figure 6 illustrates the categorisation of various factors into correlated dimensions. Again, *general* and *extended knowledge* are among the first indicators to cluster into a single dimension. The same is observed for *transaction* and *saving* as the prominent factors of *usage. Planning* does not load clearly on within any dimension. When the number of dimensions is increased to six, *awareness* loses its sole dimension in favour of *planning*. *Access* also loses its sole dimension, joining planning with a negative correlation. However, all other dimensions remain largely intact.

Further reduction in the number of dimensions causes *numeracy* to join the financial literacy group, but there is little change in the remaining dimensions. Here, five latent dimensions are, interestingly, very similar to the conceptually classified dimensions. The first dimension is financial literacy, with *awareness* left out. The second dimension is risk management. The third dimension encompasses the primary factors of *usage*, while *planning* and *accounting* are often overlooked. The last two dimensions can also be interpreted as literacy-related.

Statistically, it is not feasible to reduce the dimensions any further while maintaining all the contributing factors. Nevertheless, the construction of the four dimensions reflects the strong stance of the conceptual dimensions of *financial literacy* and *usage*. The FA results provide a qualified answer to RQ1 (the five-dimensional conceptual structure is recognisable in the data but not fully separable) and, with respect to RQ3, identify literacy and usage as the constructs whose boundaries most require refinement.

## Discussion

The statistical analyses validate a multidimensional structure of financial behaviour, offering a refined conceptual framework for future research. Based on the findings, a model can be proposed that views financial behaviour as an ecosystem of interrelated constructs. This framework can be summarised as follows:

Foundational Layer (Prerequisite): Financial inclusion acts as a static gateway. It is a necessary condition for participation, but does not, on its own, drive the nature or quality of that participation.

Cognitive and Perceptual Core (Enablers): Financial literacy and trust form the core cognitive and perceptual engine. Literacy provides the knowledge to navigate the system, while Trust provides the confidence to engage with it. The findings suggest that literacy is multifaceted, with its components (e.g., numeracy, knowledge) contributing differently across the model.

Action Layer (Manifestation): Usage represents the primary manifestation of behaviour, where knowledge and trust are translated into tangible actions such as *transaction* and *saving*.

Strategic Layer (Application): Risk management shows some separation from usage, but evidence of a distinct advanced dimension is exploratory and should be interpreted with caution. It is not about the quantity of use, but rather the quality and complexity of financial decision-making, which serves as an application of literacy and trust under uncertain conditions.

This structure moves beyond a linear, literacy-driven model, providing a more holistic map for researchers and practitioners. The results from the statistical analyses (correlation matrix, PCA, and FA) offer a comprehensive understanding of the multidimensionality of financial behaviour. These findings provide crucial insights into the relationships between the dimensions of financial inclusion, literacy usage, trust, and risk management. They also underscore the complexity of financial behaviours, highlighting the interconnectedness and independence of the conceptual dimensions. The following sections discuss the specific interdependencies and implications of this framework in detail.

### Correlations: interdependencies and disparities

The correlation analysis reveals both expected and surprising relationships among the dimensions of financial behaviour, reflecting the intricate dynamics of financial decision-making. The moderate positive correlation between financial literacy and usage is consistent with the literature. Financial literacy enables individuals to navigate financial systems effectively, engage with financial products, and make informed decisions (Lusardi et al. 2017). This relationship underscores the crucial role of financial education in promoting active financial participation, particularly among populations with access to a range of financial services.

Similarly, the modest positive correlation between financial inclusion and usage is consistent with the idea that access may support financial activity. However, the correlation alone does not establish a prerequisite status. The lack of correlation between access and advanced financial behaviours, such as *planning* and *accounting*, further suggests that inclusion alone is insufficient. This finding aligns with critiques that financial inclusion policies focusing solely on access fail to translate into meaningful financial engagement (Beck et al. 2009; Sarma and Pais 2011). These results highlight the need for complementary initiatives, such as financial literacy programmes and trust-building efforts, to bridge the gap between access and active usage.

The relationship between trust and financial literacy also emerges as a key finding with practical implications. Trust in financial institutions fosters confidence, which, when combined with financial literacy, can drive greater adoption of financial products and services (Guiso et al. 2008). This relationship underscores the importance of integrating educational initiatives with trust-building measures, particularly in contexts where institutional transparency is lacking.

The near-zero correlation between usage and risk challenges the assumption that greater financial engagement necessarily leads to riskier behaviours. Instead, it suggests that, in this dataset, usage is not meaningfully associated with openness to riskier financial products. This finding aligns with behavioural finance theories, such as prospect theory, which argue that individuals are risk-averse when faced with potential losses (Kahneman and Tversky 1979). It also emphasises the importance of understanding the relationship between financial literacy, usage, and risk management.

Financial literacy emerges as a pivotal factor across multiple dimensions, while financial inclusion plays a more limited role, beyond facilitating use. The negligible relationship between *usage* and *risk* indicates that increased engagement does not inherently lead to riskier financial behaviours, with important implications for financial service providers aiming to encourage responsible use.

From a theoretical perspective, these findings support the Financial Capability Framework, as financial behaviours are influenced by a combination of knowledge, access, and psychological factors (Sherraden 2013). The strong correlations between financial literacy sub-dimensions and various financial behaviours underscore the importance of comprehensive financial education that extends beyond basic knowledge to encompass advanced concepts and applications.

### PCA and the multidimensionality of financial behaviour

The contributions of all five conceptual dimensions to the first latent dimension, particularly the dominant roles of *literacy* and *usage*, highlight the centrality of these factors in explaining variance within financial behaviour. The connections between *literacy* and *usage* reflect the intertwined nature of knowledge and action, with informed individuals more likely to engage actively with financial services.

The near-independence of inclusion, trust, and risk in their respective dimensions suggests that these concepts capture distinct aspects of financial behaviour. However, the overlap between *literacy* and *usage* across multiple latent dimensions points to the complexity of these constructs. The spread of financial literacy factors, including general and specialised knowledge, across three dimensions underscores its multifaceted nature. This complexity underscores the importance of treating financial literacy as a multidimensional construct that encompasses basic knowledge, awareness, and advanced competencies.

The alignment of latent dimensions with conceptual classifications validates the robustness of the proposed framework. However, the observed overlaps and correlations between certain factors, such as numeracy and risk, or awareness and planning, suggest opportunities to refine the framework to capture the interconnectedness of financial behaviours better.

The PCA results depicted in Figs. 3 and 4 provide valuable insights into the validated structure of our financial behaviour model. The analysis shows that the Financial Literacy construct is structurally central, sharing significant variance with multiple other dimensions, particularly *usage*. In contrast, Financial Inclusion appears as a more isolated construct, primarily linked to *usage*. The dominance of *literacy* and *usage* in the principal components suggests these two constructs are the most significant sources of variation within the overall financial behaviour framework. The PCA and correlation results are consistent with an association between literacy and usage, while usage shows no meaningful association with risk-taking in the present data.

### FA and latent structures

The dominance of literacy and usage in the factor solutions suggests that they are foundational precursors that future studies should test as predictors of actual financial behaviour. The partial convergence of trust and risk at lower factor counts suggests an affinity between the two, reflecting trust’s role in mitigating perceived risks and enabling confident decision-making. However, the three-factor solution indicates only a partial association rather than a clearly merged factor.

The FA results depicted in Fig. 6 further clarify the model’s latent structure. The analysis empirically separates constructs such as *financial literac*y, *risk management*, and *trust* into distinct statistical factors, providing strong support for the proposed multidimensionality of financial behaviour. The strong loadings of general and extended knowledge variables onto a single factor validate the internal coherence of the *financial literacy* construct. Similarly, the emergence of distinct factors in *risk management* and *trust* supports their conceptualisation as separate yet related dimensions within the overall framework. This structural evidence suggests that interventions might be most effective when targeted at these distinct dimensions.

### Implications for theory, practice, and policy

The findings from the statistical analyses have several implications for research, policy, and practice:

Redefining Financial Inclusion: The lack of correlation between inclusion and advanced financial behaviours underscores the need to move beyond access-focused inclusion policies. Efforts should focus on enabling meaningful engagement through complementary initiatives, such as financial literacy programs and trust-building measures.

Multidimensional Financial Literacy: The spread of literacy factors across multiple dimensions highlights the need to treat financial literacy as a multidimensional construct. Educational programmes should address both foundational knowledge and advanced competencies, tailored to the needs and contexts of different populations.

Trust as a Catalyst: The correlation between trust and literacy emphasises the importance of building confidence in financial systems. This can be achieved through transparency initiatives, consumer protection measures, and targeted educational campaigns. For financial services marketers, the same finding speaks to segmentation and communication strategies that treat consumers in developing markets as heterogeneous rather than uniform: evidence from sub-Saharan Africa shows that trust, alongside the availability, accessibility, and affordability of services, differentially drives service selection across consumer segments (Chikweche et al. 2024), and trust-based relationships can be strategically leveraged to build customer loyalty (Sholevar and Bachmann 2025).

Understanding Usage and Risk: The near-zero correlation between *usage* and *risk* suggests that financial engagement does not necessarily lead to risk-taking. This finding highlights the importance of distinguishing between breadth (frequency) and depth (complexity) of financial engagement in research and practice.

Survey design: Large-scale financial surveys can adopt a similar model to pinpoint the critical dimensions when designing future instruments based on prior ones, rather than relying solely on questions and factors that make conceptual sense.

Framework Refinement: The overlaps and correlations observed in PCA and FA suggest opportunities to refine the conceptual framework by incorporating subcategories or hybrid dimensions to capture the complexity of financial behaviour better.

### A hypothesis-generating framework for future research

The primary value of this validated structural framework lies in its ability to generate specific, testable hypotheses for future research aimed at establishing causal relationships. The primary objective of this work is to allow the latent dimensions to reveal their interdimensional dependencies, rather than to test common-sense assumptions statistically. Based on the exploratory findings, a theoretical model is proposed to describe the plausible mechanisms linking these dimensions. This model, which requires future empirical testing for causality, posits the following pathways:

Hierarchical pathway of financial behaviour: Financial inclusion serves as a prerequisite, providing the infrastructure for financial participation (access to inclusion). Financial literacy is posited to enhance the ability to navigate financial systems and make informed choices, but, in isolation, it does not lead to sound decisions without contextual and behavioural integration (literacy as an enabler). Usage is conceptualised as the activation of inclusion and literacy, translating capabilities into practical engagement with financial services (usage as engagement). Trust is expected to moderate engagement by reducing perceived barriers. In contrast, risk management is expected to balance opportunities and vulnerabilities, thereby moderating the relationship between trust and engagement (trust and risk management as moderators).

Progressive nature of dimensions: Dimensions such as financial literacy exhibit a progressive structure, in which foundational knowledge (general literacy) leads to advanced competencies (extended knowledge), enabling more complex behaviours. Financial literacy shows the clearest progressive pattern through the relationship between general and extended knowledge. In contrast, the present data do not show a simple progression from access to more sophisticated practices such as planning or accounting.

Interdependency and feedback loops: Interdependencies between dimensions (e.g., literacy enabling usage, trust influencing risk management) may create feedback loops in which improvement in one dimension reinforces others. For example, literacy, trust, and usage may reinforce one another over time, but the present cross-sectional correlations show only modest associations and do not establish a feedback loop.

From this model, the following hypotheses are proposed. They were generated by the exploratory construct validation reported above and were not tested with behavioural outcome data in the present study:

H1: Financial inclusion is a necessary but insufficient condition for active financial behaviour; its effectiveness is mediated by financial literacy and trust.

H2: Financial literacy has a multidimensional structure, where general knowledge and numeracy serve as prerequisites for advanced competencies.

H3: Usage of financial services is positively associated with financial literacy but not associated with risk-taking propensity.

H4: Trust in financial systems moderates the relationship between financial inclusion and usage, particularly in contexts with low institutional transparency.

H5: Risk management is a distinct dimension that influences the complexity and type of financial decisions rather than the frequency of financial service use.

## Conclusion

This study achieves its stated objectives of (i) specifying a five-pillar conceptualisation of financial behaviour, and (ii) validating that structure statistically in an exploratory manner; it intentionally does not test behavioural outcomes, i.e., the next research step. By treating these dimensions as interrelated components of a broader construct, the relationships among them become clearer, enabling a deeper understanding of their individual and collective contributions to financial behaviour.

Financial behaviour is theorised as a multidimensional construct, potentially shaped by distinct yet interconnected dimensions, including financial inclusion, literacy, usage, trust, and risk management. These dimensions operate synergistically to influence decision-making and behaviour, reflecting dynamic interactions between individual capabilities, societal structures, and institutional contexts. Financial behaviour emerges from multidimensional interactions: foundational factors (e.g., financial inclusion) enable access; cognitive factors (e.g., literacy) drive understanding; behavioural factors (e.g., usage) manifest engagement; and perceptual factors (e.g., trust and risk management) guide decision-making under uncertainty.

The study offers three key implications for scholars and practitioners. First, reframing the construct space: by providing exploratory evidence that several theoretically defined pillars co-exist while remaining partly overlapping, this study moves the discourse beyond the single-driver financial-literacy paradigm that has dominated since Lusardi and Mitchell (2011). Second, validating, not presuming, dimensionality: PCA and FA provide partial convergence with the conceptual map, especially around literacy and usage, but the structure remains exploratory and does not fully separate all proposed dimensions. This dual-technique validation demonstrates that dimensional claims in personal finance research can and should be tested rather than assumed. Third, context-specific instrumentation: the purpose-built Ethiopian dataset demonstrates that fine-grained, local instruments can uncover features overlooked by global omnibus surveys, establishing a template for future construct-validation studies in other under-researched settings.

Together, these insights offer a framework that can reshape how researchers model and how policymakers target the antecedents of financial behaviour. The constructs validated here form a ready-made framework for longitudinal or experimental work that links them to concrete behavioural outcomes, such as saving trajectories, credit performance, or portfolio diversification.

### Limitations

Data scope: Although the original data collected for this study are tailored to the research objectives, they are limited to a specific geographic and demographic context. Generalisability to other populations and regions remains a challenge and requires further validation.

Cross-study comparisons: The framework’s applicability is constrained by the lack of independent analyses using different datasets. Comparative studies are necessary to test the framework’s reliability and robustness across diverse contexts.

Complexity of dimensions: Some dimensions, particularly financial literacy, are highly complex and overlap with other dimensions. Future research should refine these constructs to capture their multifaceted nature better.

Data collection: Since this research aimed to collect data from a diverse and understudied population, sub-Saharan Africa was chosen to provide a more heterogeneous picture of financial behaviour. This came with its own challenges as questionnaire literacy and the diversity of regional languages made interviewer-assisted administration essential, and the dialogue between participants and interviewers could introduce inevitable bias despite the safeguards adopted.

Sample size and multidimensionality: A critical point to stress is that the data (e.g., correlations or KMO tests) are statistically acceptable, but they do not necessarily reflect strong, decisive relationships. There are two key implications. First, the multidimensional nature of financial behaviour makes it difficult to draw definitive conclusions. This is indeed the same point the present paper aims to address: the drawbacks of oversimplifying multifaceted financial behaviour. Second, to inspect multidimensional features, large datasets are typically required. Unfortunately, it is often not feasible to conduct such large surveys within individual research projects. This is why many researchers rely on secondary data from large, general surveys, which often lose their multidimensional nature due to their generality and lack of targeted focus on interconnected dimensions.

### Future research

The most immediate step is confirmatory: the hypotheses (H1-H5) should be tested through longitudinal and experimental studies designed to establish causality, and the factor structure should be re-examined using confirmatory factor analysis, replication with independent datasets, and linkage to external behavioural outcomes. Future research should also explore additional dimensions (e.g., self-efficacy and financial well-being) and examine how these dimensions evolve and interact over time. Beyond these steps, three extensions appear especially promising.

Dynamic modelling: future iterations of the framework can incorporate dynamic models, such as system dynamics or agent-based simulations, to capture the complex feedback loops and interactions between dimensions.

Behavioural insights: integrating behavioural finance principles, such as cognitive biases and emotional drivers, can enrich the framework, providing a deeper understanding of deviations from rational financial behaviour.

Cultural and contextual variations: expanding the framework to account for these variations can enhance its applicability, particularly when addressing the unique challenges faced by low-income populations or regions with low levels of institutional trust.

## Supplementary Information

Below is the link to the electronic supplementary material.

Supplementary Material 1

Supplementary Material 2

Supplementary Material 3

Supplementary Material 4

Supplementary Material 5

Supplementary Material 6

Supplementary Material 7

Supplementary Material 8

Supplementary Material 9

Supplementary Material 10

Supplementary Material 11

## Appendix

See Table 2.

**Table 2:** Questions associated with the dimensions and their underlying factors

Question	Dimension	Factor
Do you have insurance?	Inclusion	Access
Do you have an account in a state bank?	Inclusion	Access
Do you have an account in a private bank	Inclusion	Access
Do you have a regular income?	Inclusion	Means
Does your income cover living expenses, if not, what do you do?	Inclusion	Means
Is it possible to open a bank account with less than 100 birr	Literacy	Awareness
Do you know what is cryptocurrency?	Literacy	Extended Knowledge
Do you know anything about the stock market in Ethiopia?	Literacy	Extended Knowledge
level of knowledge about Mobile money	Literacy	Extended Knowledge
level of knowledge about Digital payment	Literacy	Extended Knowledge
level of knowledge about Credit card	Literacy	Extended Knowledge
level of knowledge about M-birr	Literacy	Extended Knowledge
level of knowledge about CBE-Birr	Literacy	Extended Knowledge
level of knowledge about instalment	Literacy	General Knowledge
level of knowledge about Savings account	Literacy	General Knowledge
level of knowledge about Microfinance loan	Literacy	General Knowledge
level of knowledge about Mortgage	Literacy	General Knowledge
level of knowledge about Life insurance	Literacy	General Knowledge
level of knowledge about Weather insurance	Literacy	General Knowledge
level of knowledge about Current account	Literacy	General Knowledge
Assume you deposited 1000 birr in a savings account with 5% interest rate per month. After a year, how much money you would have if you do not withdraw or add any money to your account?	Literacy	Numeracy
If it is possible, do you save money as cryptocurrency?	Risk	Openness
If it is possible, do you invest money in the stock market?	Risk	Openness
How much do you trust your bank?	Trust	Broad scope Trust
How much do you trust the banking system?	Trust	Broad scope Trust
How much do you trust your financial adviser or the bank officer?	Trust	Narrow scope Trust
Would you trust a bank from the other regions such as Amhara?	Trust	National Trust
Will you recommend your bank to your family or friends?	Trust	Net Promoter Score
Do you think the bank officers trust you?	Trust	Reciprocal Narrow scope Trust
How would you rate the quality of banking services you currently use?	Trust	Satisfaction
Do you trust ATM or electronic banking?	Trust	Technological Trust
Do you trust mobile money?	Trust	Technological Trust
How much do you feel well-informed about bank products, services and charges?	Trust	Transparency
Do you keep track of your expenditure?	Usage	Accounting
What is your plan for retirement?	Usage	Planning
In the past 12 months, did you save any money?	Usage	Saving
How did you save money?	Usage	Saving
Have you ever transferred money?	Usage	Transaction
How did you do it?	Usage	Transaction
In the past 12 months, did you send any money to other people?	Usage	Transaction
How did you send money?	Usage	Transaction
In the past 12 months, did you receive any money from other people?	Usage	Transaction
How did you receive money?	Usage	Transaction
