# Supplementary material for: Latent dimensions of financial behaviour derived by principal component and factor analysis
Source: J Financ Serv Mark. 2026 Aug 7;31(3):50. doi: 10.1057/s41264-026-00384-5 (PMC13584979; doi:10.1057/s41264-026-00384-5)
Supplement: Supplementary file 1 [file 41264_2026_384_MOESM10_ESM.docx]

# Table 6. Questionnaire (Demographic questions G1 to G10)

| **G1** | **Category of respondents** |
| --- | --- |
| **1** | Employee |
| **2** | Owner-manager of micro enterprise |
| **3** | Owner-manager of small enterprise |

| **G2** | **What is your gender?** |
| --- | --- |
| **1** | Male |
| **2** | Female |

| **G3** | **What is your age?** |
| --- | --- |
| **1** | 18-19 |
| **2** | 20-29 |
| **3** | 30-39 |
| **4** | 40-49 |
| **5** | 50-59 |
| **6** | 60-69 |

| **G4** | **What is your marital status?** |
| --- | --- |
| **1** | Single |
| **2** | Married/Living with partner |

| **G5** | **Which area do you live in? (address, Phone)** |
| --- | --- |

| **G6** | **What is your religion?** |
| --- | --- |
| **1** | Muslim |
| **2** | Christian |
| **3** | Others |

| **G7** | **What is your highest education level?** |
| --- | --- |
| **1** | Primary and secondary schools only |
| **2** | High school |
| **3** | Junior college/college of technology |
| **4** | Undergraduate |
| **5** | Masters degree and above |
| **6** | No formal education, but able to read and write |
| **7** | Completely illiterate |

| **G8** | **What is your employment status? (multiple choices)** |
| --- | --- |
| **1** | Company employee |
| **2** | Government employee |
| **3** | Farmer |
| **4** | Private business- Specify please |
| **5** | Part-timer- Specify please |
| **6** | House maid |
| **7** | Student |
| **8** | Unemployed |
| **9** | Other- Please specify |

| **G9** | **Do you have any assets? (multiple choices)** |
| --- | --- |
| **1** | Savings |
| **2** | Livestock- Specify |
| **3** | House |
| **4** | Farm |
| **5** | Land |
| **6** | Machinery |
| **7** | Other- Please specify |

| **G10** | **Are you head of the household?** |
| --- | --- |
| **1** | Yes |
| **2** | No |

**Financial Literacy and Inclusion Questionnaire (QL1 to QL38).**

| **QL1** | **Do you have a regular income?** |
| --- | --- |
| **1** | Yes |
| **2** | No |

| **QL2** | **what is your average monthly income in Birr** |
| --- | --- |
| **1** | 0-1,000 |
| **2** | 1001-5,000 |
| **3** | 5,001-10,000 |
| **4** | 10,001-15,000 |
| **5** | 15,001-20,000 |
| **6** | >20,000 |
| **7** | I don't want to say |

| **QL3** | **If No, What are the reasons for your answer ?** |
| --- | --- |
| **1** | I don't have a job |
| **2** | I don't have enough money to start a job |
| **3** | My income is not predictable |
| **4** | I am a student |
| **5** | I don't want to say |

| **QL4** | **Do you keep track of your expenditure?** |
| --- | --- |
| **1** | Yes |
| **2** | No |

| **QL5** | **Does your income cover living expenses, if not, what do you do? (multiple choices)** |
| --- | --- |
| **1** | Yes |
| **2** | No, Cut down expenses |
| **3** | No, Borrow money from friends and family |
| **4** | No, Spend our savings |
| **5** | No, use a credit card |
| **6** | No, Sell our financial assets |
| **7** | No, work extra hours |
| **8** | No, Others |

| **QL6** | **If you lose your income, how long your assets or saving would last to meet your living expenses?** |
| --- | --- |
| **1** | Less than a week |
| **2** | More than one week but less than a month |
| **3** | Between one month to three months |
| **4** | More than three months |
| **5** | I don't know |

| **QL7** | **How do you spend your money? (On a scale of 1 to 5, what is the rating for the following options)** |
| --- | --- |
| **1** | Living expenses |
| **2** | Paying children's educational expenses |
| **3** | Saving for emergency |
| **4** | Buying a House /Farm/Sheep/Ox |
| **5** | Saving for Retirement |

| **QL8** | **If you have any money left, what do you do? (On a scale of 1 to 5, what is the rating for the following options)** |
| --- | --- |
| **1** | Keep it in cash |
| **2** | Deposit in bank |
| **3** | Spending on clothes, foods, |
| **4** | Buying a House /Farm/Sheep/Ox |
| **5** | Save in the savings club |

| **QL9** | **Assume you deposited 1000 birr in a savings account with 5% interest rate per month. After a year, how much money you would have if you don't withdraw or add any money to your account?** |
| --- | --- |
| **1** | More than 1500 |
| **2** | Less than 1500 |
| **3** | Exactly 1500 |
| **4** | Don't know |

| **QL10** | **On a scale of 1 to 5, how much do you know about the following products?** |
| --- | --- |
| **1** | Instalment |
| **2** | Savings account |
| **3** | Mobile money |
| **4** | Digital payment |
| **5** | Credit card |
| **6** | Microfinance loan |
| **7** | M-birr |
| **8** | Mortgage |
| **9** | Life insurance |
| **10** | Weather insurance |
| **11** | Current account |
| **12** | CBE-Birr |

| **QL11** | **What do you think is the current inflation rate?** |
| --- | --- |
| **1** | Under 5% |
| **2** | 5-10% |
| **3** | 11-20% |
| **4** | 21-30% |
| **5** | 31-40% |
| **6** | I don't know |

| **QL12** | **Imagine you have 2000br now. After one year, how much would you be able to buy with this money?** |
| --- | --- |
| **1** | More than today |
| **2** | Exactly the same |
| **3** | Less than today |
| **4** | Do not know |

| **QL13** | **What is your plan for retirement? (multiple choice)** |
| --- | --- |
| **1** | Government pension |
| **2** | Employment pension plan |
| **3** | Sale of financial assets |
| **4** | Sale of non-financial assets |
| **5** | Relying on a spouse/partner children or other family members for support |
| **6** | Other (please specify) |
| **7** | No plan |

| **QL14** | **Is it possible to open a bank account with less than 100 birr** |
| --- | --- |
| **1** | Yes |
| **2** | No |
| **3** | I don't know |

| **QL15** | **How do you pay your water and electric bill? (multiple choices)** |
| --- | --- |
| **1** | By cash in the bank |
| **2** | By mobile money- (CBE-Birr- M-birr) |
| **3** | By ATM |
| **4** | I don't pay any bills |
| **5** | Other (please specify) |

| **QL16** | **Have you ever transferred money?** |
| --- | --- |
| **1** | Yes |
| **2** | No |

| **QL17** | **How did you do it?(multiple choices)** |
| --- | --- |
| **1** | By Bank |
| **2** | By ATM |
| **3** | By Western Union |
| **4** | By friends and family |
| **5** | By CBE-birr |
| **6** | All of them |
| **7** | Other (please specify) |

| **QL18** | **If no, What are the reasons for your answer?(multiple choices)** |
| --- | --- |
| **1** | I don't have enough money to transfer |
| **2** | I don't have anyone to transfer |
| **3** | I don't have bank account |
| **4** | I don't trust bank with my money |
| **5** | All of them |
| **6** | Other (please specify) |

| **QL19** | **Do you have insurance?** |
| --- | --- |
| **1** | Yes |
| **2** | No |

| **QL20** | **What kind of insurance do you have?** |
| --- | --- |
| **1** | Life Insurance |
| **2** | Car insurance |
| **3** | Weather insurance |
| **4** | Health insurance |
| **5** | All of them |
| **6** | Other (please specify) |

| **QL21** | **If no, What are the reasons for your answer?(multiple choices)** |
| --- | --- |
| **1** | I don't have enough money to buy insurance |
| **2** | I don't have any valuable thing to buy insurance |
| **3** | I don't trust insurance companies |
| **4** | I don't understand insurance and its mechanism. |
| **5** | All of them |
| **6** | Other (please specify) |

| **QL22** | **Do you know what is cryptocurrency?** |
| --- | --- |
| **1** | Yes |
| **2** | No |

| **QL23** | **Do you know anything about the stock market in Ethiopia?** |
| --- | --- |
| **1** | Yes |
| **2** | No |

| **QL24** | **In the past 12 months, did you save any money?** |
| --- | --- |
| **1** | Yes |
| **2** | No |

| **QL25** | **How did you save money? (multiple choices)** |
| --- | --- |
| **1** | In the bank account |
| **2** | In savings clubs |
| **3** | Lent it to friends and family |
| **4** | Bought financial assets |
| **5** | All of them |
| **6** | Others (please specify) |

| **QL26** | **If NO, please explain it (multiple choices)** |
| --- | --- |
| **1** | I did not have enough money to save. |
| **2** | I did not have a bank account. |
| **3** | I don't know how to save money |
| **4** | I prefer to spend my all money instead of saving |
| **5** | All of them |
| **6** | Others (please specify) |

| **QL27** | **In the past 12 months, did you send any money to other people?** |
| --- | --- |
| **1** | Yes |
| **2** | No |

| **QL28** | **How did you send money? (multiple choices)** |
| --- | --- |
| **1** | Through the bank |
| **2** | Through friends and family |
| **3** | Through mobile money- CBE-birr |
| **4** | Through western union |
| **5** | All of them |
| **6** | Others (please specify) |

| **QL29** | **If NO, please explain it (multiple choices)** |
| --- | --- |
| **1** | I did not have enough money to send. |
| **2** | I did not have anyone to send money. |
| **3** | I don't know how to send money |
| **4** | I don't like giving my money to others. |
| **5** | All of them |
| **6** | Others (please specify) |

| **QL30** | **In the past 12 months, did you receive any money from other people?** |
| --- | --- |
| **1** | Yes |
| **2** | No |

| **QL31** | **How did you receive money? (multiple choices)** |
| --- | --- |
| **1** | Through the bank |
| **2** | Through friends and family |
| **3** | Through mobile money |
| **4** | Through western union |
| **5** | All of them |
| **6** | Others (please specify) |

| **QL32** | **If NO, please explain it (multiple choices)** |
| --- | --- |
| **1** | I did not have anyone to send me money. |
| **2** | I don't know how to receive money |
| **3** | I don't have a bank account |
| **4** | It is not easy and troubling. |
| **5** | All of them |
| **6** | Others (please specify) |

| **QL33** | **If it is possible, do you save money as cryptocurrency?** |
| --- | --- |
| **1** | Yes |
| **2** | No |

| **QL34** | **If YES, please explain it (multiple choices)** |
| --- | --- |
| **1** | I trust it |
| **2** | I found it easy to understand and use it |
| **3** | It is financially rewarding |
| **4** | It is innovative |
| **5** | All of them |
| **6** | Others (please specify) |

| **QL35** | **If NO, please explain it (multiple choices)** |
| --- | --- |
| **1** | I do not trust it. |
| **2** | It is difficult to understand. |
| **3** | I do not have access to online banking. |
| **4** | I do not have enough knowledge about it |
| **5** | All of them |
| **6** | Others (please specify) |

| **QL36** | **If it is possible, do you invest money in the stock market?** |
| --- | --- |
| **1** | Yes |
| **2** | No |

| **QL37** | **If YES, please explain it (multiple choices)** |
| --- | --- |
| **1** | I trust it |
| **2** | I found it easy to understand and use it |
| **3** | It is financially rewarding |
| **4** | It is innovative |
| **5** | All of them |
| **6** | Others (please specify) |

| **QL38** | **If NO, please explain it (multiple choices)** |
| --- | --- |
| **1** | I do not trust it. |
| **2** | It is difficult to understand. |
| **3** | I do not have access to internet |
| **4** | I do not have enough knowledge about it |
| **5** | All of them |
| **6** | Others (please specify) |

**Trust Questionnaire (QT1 to QT28).**

| **QT1** | **On a scale of 1 to 5, how much do you trust your bank?** |
| --- | --- |

| **QT2** | **On a scale of 1 to 5, how much do you trust the banking system?** |
| --- | --- |

| **QT3** | **On a scale of 1 to 5, how much do you trust your financial adviser or the bank officer?** |
| --- | --- |

| **QT4** | **On a scale of 1 to 5, how much do you feel well-informed about bank products, services and charges?** |
| --- | --- |

| **QT5** | **On a scale of 1 to 5, how much do you trust a bank from the other regions such as Amhara?** |
| --- | --- |

| **QT6** | **On a scale of 1 to 5, how would you rate the quality of banking services you currently use?** |
| --- | --- |

| **QT7** | **What do you mean when you say you trust a financial organisation (multiple choice)** |
| --- | --- |
| **1** | My money is safe with them |
| **2** | The organisation is reliable. |
| **3** | My transactions are secure. |
| **4** | My transactions are timely. |
| **5** | The fees are reasonable. |
| **6** | I can access my money whenever I need it. |
| **7** | The process and procedures are transparent. |
| **8** | My data and information are protected. |
| **9** | My money would be guaranteed. |
| **10** | All of them. |
| **11** | Other (please name it) |

| **QT8** | **Do you have an account in Private owned bank or State-owned bank?** |
| --- | --- |
| **1** | State owned bank |
| **2** | Private owned bank |
| **3** | Both |

| **QT9** | **Do you trust State-owned/Private owned banks?** |
| --- | --- |
| **1** | I trust State-owned banks but not Private owned banks. (please answer questions 10-11) |
| **2** | I trust Private owned banks but not State-owned banks. (please answer questions 12-13) |
| **3** | I do not trust either of them. (please answer question 14) |
| **4** | I trust both, Private owned and State-owned banks. (please answer question 15) |
| **5** | I don't know |

**What are the reasons for your answer to question 9?**

| **QT10** | **I trust State owned banks because: (multiple choices)** |
| --- | --- |
| **1** | They do not stay away when I have problems. |
| **2** | They will not go bankrupt. |
| **3** | They treat customers fairly. |
| **4** | They are more competent than Private owned banks. |
| **5** | The charges and fees are reasonable. |
| **6** | The costs and risks are clear. |
| **7** | They are regulated and follow the law and regulations. |
| **8** | They are government owned. |
| **9** | They respond to the customers' complaints quickly. |
| **10** | They are good financial advisers. |
| **11** | All of Them |
| **12** | Other (please name it). |

| **QT11** | **I do not trust Private owned banks because: (multiple choices)** |
| --- | --- |
| **1** | They stay away when I have a problem |
| **2** | They might go bankrupt. |
| **3** | They do not treat customers fairly. |
| **4** | They are not competent. |
| **5** | They charge unreasonably. |
| **6** | The costs and risks are not clear. |
| **7** | They are not regulated. |
| **8** | They are not government-owned. |
| **9** | They do not respond to the customers' complaints quickly. |
| **10** | I do not trust their financiaadvicees. |
| **11** | All of Them |
| **12** | Other (please name it) |

| **QT12** | **I trust Private owned banks because: (multiple choices)** |
| --- | --- |
| **1** | They do not stay away when I have problems |
| **2** | They will not go bankrupt. |
| **3** | They treat customers fairly. |
| **4** | They are more competent than other banks. |
| **5** | The charges and fees are reasonable. |
| **6** | The costs and risks are clear. |
| **7** | They are regulated and follow the law and regulations. |
| **8** | They are not government-owned. |
| **9** | They respond to the customers' complaints quickly. |
| **10** | They are good financial advisers. |
| **11** | All of Them |
| **12** | Other (please name it). |

| **QT13** | **I do not trust State-owned banks because: (multiple choices)** |
| --- | --- |
| **1** | They stay away when I have a problem |
| **2** | They might go bankrupt. |
| **3** | They do not treat customers fairly. |
| **4** | They are not competent. |
| **5** | They charge unreasonably. |
| **6** | The costs and risks are not clear. |
| **7** | They are not regulated. |
| **8** | They are government owned |
| **9** | They do not respond to the customers' complaints quickly. |
| **10** | I do not trust their financial advice. |
| **11** | All of Them |
| **12** | Other (please name it) |

| **QT14** | **I do not trust either of them because: (multiple choices)** |
| --- | --- |
| **1** | They stay away when I have a problem |
| **2** | They might go bankrupt. |
| **3** | They do not treat customers fairly. |
| **4** | They are not competent. |
| **5** | They charged unreasonably. |
| **6** | The costs and risks are not clear. |
| **7** | They are not regulated. |
| **8** | They do not respond to the customers' complaints quickly. |
| **9** | They are not honest with customers. |
| **10** | I do not trust their financial advice. |
| **11** | All of Them |
| **12** | Other (name it please) |

| **QT15** | **I trust both of them because: (multiple choices)** |
| --- | --- |
| **1** | They do not stay away when I have problems |
| **2** | They will not go bankrupt. |
| **3** | They treat customers fairly. |
| **4** | They are more competent than other banks. |
| **5** | The charges and fees are reasonable. |
| **6** | The costs and risks are clear. |
| **7** | They are regulated and follow the law and regulations. |
| **8** | They respond to the customers' complaints quickly. |
| **9** | They are honest with customers. |
| **10** | They are good financial advisers. |
| **11** | All of Them |
| **12** | others (name it please). |

| **QT16** | **Will you recommend your bank to your family or friends?** |
| --- | --- |
| **1** | Yes |
| **2** | No |

| **QT17** | **If YES, please explain it (multiple choices)** |
| --- | --- |
| **1** | This bank is financially strong |
| **2** | This brand has many branches around the Ethiopia |
| **3** | They provide good services |
| **4** | Working with this bank is financially rewarding. |
| **5** | They are efficient with regards to time and cost. |
| **6** | They are trustworthy |
| **7** | They have a good customer relationship management. |
| **8** | They are more competent than the other banks. |
| **9** | All of them |
| **10** | Other (please name it) |

| **QT18** | **If NO, please explain it (multiple choices)** |
| --- | --- |
| **1** | It is risky, they might go bankrupt. |
| **2** | They don't have many branches around Ethiopia. |
| **3** | They do not provide good services. |
| **4** | Working with this bank is not financially rewarding. |
| **5** | They are not efficient with regards to time and cost. |
| **6** | They are not trustworthy. |
| **7** | They do not have a good customer relationship management. |
| **8** | They are not competent. |
| **9** | All of them |
| **10** | Other (please name it) |

| **QT19** | **Do you trust ATM or electronic banking?** |
| --- | --- |
| **1** | Yes |
| **2** | No |

| **QT20** | **If YES, please explain it (multiple choices)** |
| --- | --- |
| **1** | My money is safe with them. |
| **2** | Online banking is accessible. |
| **3** | Banks are always crowded. |
| **4** | I have a problem with opening times of the bank |
| **5** | By online banking and ATM, I can get better information and services. |
| **6** | The government protects my electronic money. |
| **7** | All of them |
| **8** | Others (please name it) |

| **QT21** | **If NO, please explain it (multiple choices)** |
| --- | --- |
| **1** | I do not trust online banking with my money |
| **2** | It is not accessible from anywhere at anytime. |
| **3** | Most of the time, ATMs do not have money. |
| **4** | ATMs do not provide me all the services that I need. |
| **5** | I prefer talking with banking officers. |
| **6** | The government protects my money. |
| **7** | All of them |
| **8** | Other (please name it) |

| **QT22** | **Who does provide your mobile banking service?** |
| --- | --- |
| **1** | A bank (please name it) |
| **2** | A bank and a Mobile operator together (please name it) |
| **3** | A mobile operator (please name it) |
| **4** | Another company (please name it) |
| **5** | I don't know |

| **QT23** | **Do you trust mobile money?** |
| --- | --- |
| **1** | Yes |
| **2** | No |

| **QT24** | **If YES, please explain it (multiple choices)** |
| --- | --- |
| **1** | I trust my money with mobile money. |
| **2** | There is a legal guarantee mechanism behind the systems and services. |
| **3** | It is reliable. |
| **4** | Transactions can be done at anytime. |
| **5** | Mobile money can provide a secure transaction |
| **6** | Mobile money can provide a timely transaction |
| **7** | I trust the providers (bank or mobile operator that provides mobile banking) |
| **8** | All of them |
| **9** | Other (please name it) |

| **QT25** | **If NO, please explain it (multiple choices)** |
| --- | --- |
| **1** | I do not trust my money with mobile money. |
| **2** | There is no legal guarantee mechanism behind the systems and services. |
| **3** | It is not reliable. |
| **4** | I cannot do transaction at anytime that I need. |
| **5** | I doubt the security of transaction. |
| **6** | It takes time to make a transaction. It is not timely. |
| **7** | I don't trust the providers (bank or mobile operator that provides mobile banking) |
| **8** | All of them |
| **9** | Other (please name it) |

| **QT26** | **Do you think the bank officers trust you?** |
| --- | --- |
| **1** | Yes |
| **2** | No |
| **3** | I am just a depositor |

| **QT27** | **If YES, please explain it (multiple choices)** |
| --- | --- |
| **1** | I have a good amount of money in my account |
| **2** | I always pay my instalments on time |
| **3** | I understand the technical and banking terms and concepts |
| **4** | I have a good and profitable business |
| **5** | I work for the government |
| **6** | I am from the same region as the bank. |
| **7** | I only use the services of this bank. |
| **8** | All of them |
| **9** | Others (name it please) |

| **QT28** | **If NO, please explain it (multiple choices)** |
| --- | --- |
| **1** | I am poor |
| **2** | I cannot pay my instalments on time. |
| **3** | I do not understand the technical and banking terms and concepts |
| **4** | My income is low. |
| **5** | I do not have a job |
| **6** | I am from another region |
| **7** | I use banking services from other banks |
| **8** | All of them |
| **9** | Other (name it please) |
