# Supplementary material for: Latent dimensions of financial behaviour derived by principal component and factor analysis
Source: J Financ Serv Mark. 2026 Aug 7;31(3):50. doi: 10.1057/s41264-026-00384-5 (PMC13584979; doi:10.1057/s41264-026-00384-5)
Supplement: Supplementary file 2 [file 41264_2026_384_MOESM11_ESM.docx]

Table 7. Demography of the participants

| **Profile of respondents (total 470 individuals)** | | | |
| --- | --- | --- | --- |
| **Rural/Urban** |  | Female | Male |
|  | Urban | 78 | 71 |
|  | Rural | 157 | 164 |
| **Job category** | Employees | 74 | 79 |
|  | Micro business owners | 106 | 80 |
|  | Small business owners | 55 | 76 |
| **Age** | 18-19 | 10 | 7 |
|  | 20-29 | 100 | 97 |
|  | 30-39 | 91 | 96 |
|  | 40-49 | 27 | 20 |
|  | 50-59 | 4 | 12 |
|  | 60-69 | 3 | 3 |
| **Marital status** | Single | 69 | 80 |
|  | Married | 166 | 155 |
| **Income** | 0-1000 | 21 | 29 |
|  | 1,001-5,000 | 60 | 67 |
|  | 5,001-10,000 | 76 | 56 |
|  | 10,001-15,000 | 21 | 24 |
|  | 15,001-20,000 | 18 | 16 |
|  | >20,000 | 7 | 15 |
|  | I don't want to say | 31 | 26 |
| **Education** | Completely illiterate | 1 | 0 |
|  | No formal education | 1 | 1 |
|  | Primary and secondary | 35 | 36 |
|  | High school | 88 | 89 |
|  | Junior college | 36 | 32 |
|  | Undergraduate | 59 | 62 |
|  | Master’s degree/above | 15 | 15 |
