# Supplementary material for: Latent dimensions of financial behaviour derived by principal component and factor analysis
Source: J Financ Serv Mark. 2026 Aug 7;31(3):50. doi: 10.1057/s41264-026-00384-5 (PMC13584979; doi:10.1057/s41264-026-00384-5)
Supplement: Supplementary file 3 [file 41264_2026_384_MOESM2_ESM.docx]

Table S2a. The correlation matrix of five conceptual dimensions of financial behaviour.

| "" | "Inclusion" | "Literacy" | "Risk" | "Trust" | "Usage" |
| --- | --- | --- | --- | --- | --- |
| "Inclusion" | 1 | 0.199356387579555 | 0.0969147684078575 | 0.183940351879373 | 0.252298810307019 |
| "Literacy" | 0.199356387579555 | 1 | 0.17554606615829 | 0.234341185017028 | 0.332950384317143 |
| "Risk" | 0.0969147684078575 | 0.17554606615829 | 1 | 0.185786885577934 | 0.0128219880720788 |
| "Trust" | 0.183940351879373 | 0.234341185017028 | 0.185786885577934 | 1 | 0.144622365404894 |
| "Usage" | 0.252298810307019 | 0.332950384317143 | 0.0128219880720788 | 0.144622365404894 | 1 |
