# Supplementary material for: Latent dimensions of financial behaviour derived by principal component and factor analysis
Source: J Financ Serv Mark. 2026 Aug 7;31(3):50. doi: 10.1057/s41264-026-00384-5 (PMC13584979; doi:10.1057/s41264-026-00384-5)
Supplement: Supplementary file 4 [file 41264_2026_384_MOESM3_ESM.docx]

Table S2b. The correlation matrix of the factors of financial behaviour.

|  | Access | Means | General.Knowledge | Extended.Knowledge | Awareness | Numeracy | Openness | Accounting | Transaction | Planning | Saving |
| --- | --- | --- | --- | --- | --- | --- | --- | --- | --- | --- | --- |
| Access | 1 | -0.0366852744233387 | 0.0920032306244426 | 0.106351797961665 | 0.0195186024309667 | 0.0282016332048455 | 0.212765803917863 | -0.0578870360731834 | 0.170443730317488 | -0.164147962715859 | 0.130166825502024 |
| Means | -0.0366852744233387 | 1 | 0.155302522152255 | 0.184587602985558 | -0.0535754658377021 | -0.0398220656818189 | -0.0987196712753158 | 0.214423972993264 | 0.127835194178882 | 0.201575196606149 | 0.147307141044232 |
| General.Knowledge | 0.0920032306244426 | 0.155302522152255 | 1 | 0.530857534140204 | 0.0443353996887768 | 0.170112477257598 | -0.0321415258101506 | 0.190980946721244 | 0.241933027774689 | 0.195953477731637 | 0.120618259081156 |
| Extended.Knowledge | 0.106351797961665 | 0.184587602985558 | 0.530857534140204 | 1 | 0.0731492095944673 | 0.30438133837531 | 0.252579706013284 | 0.151366678648287 | 0.271050447484162 | 0.254255765966715 | 0.105558261011205 |
| Awareness | 0.0195186024309667 | -0.0535754658377021 | 0.0443353996887768 | 0.0731492095944673 | 1 | -0.0520058033354227 | 0.0348365927852874 | -0.0645125428231538 | 0.0151752347974812 | 0.0868579661245863 | 0.065380187020793 |
| Numeracy | 0.0282016332048455 | -0.0398220656818189 | 0.170112477257598 | 0.30438133837531 | -0.0520058033354227 | 1 | 0.113134565169438 | 0.0882895316694571 | 0.00244902625468767 | 0.0653689418600985 | 0.0234520091193245 |
| Openness | 0.212765803917863 | -0.0987196712753158 | -0.0321415258101506 | 0.252579706013284 | 0.0348365927852874 | 0.113134565169438 | 1 | -0.0202079948701305 | 0.0391153029421991 | 0.11275488287433 | -0.115315555572203 |
| Accounting | -0.0578870360731834 | 0.214423972993264 | 0.190980946721244 | 0.151366678648287 | -0.0645125428231538 | 0.0882895316694571 | -0.0202079948701305 | 1 | -0.00140579390583767 | 0.133768488183583 | 0.0202394777711096 |
| Transaction | 0.170443730317488 | 0.127835194178882 | 0.241933027774689 | 0.271050447484162 | 0.0151752347974812 | 0.00244902625468767 | 0.0391153029421991 | -0.00140579390583767 | 1 | 0.150752476820301 | 0.329817170430775 |
| Planning | -0.164147962715859 | 0.201575196606149 | 0.195953477731637 | 0.254255765966715 | 0.0868579661245863 | 0.0653689418600985 | 0.11275488287433 | 0.133768488183583 | 0.150752476820301 | 1 | 0.0701584923137728 |
| Saving | 0.130166825502024 | 0.147307141044232 | 0.120618259081156 | 0.105558261011205 | 0.065380187020793 | 0.0234520091193245 | -0.115315555572203 | 0.0202394777711096 | 0.329817170430775 | 0.0701584923137728 | 1 |
