# Supplementary material for: Latent dimensions of financial behaviour derived by principal component and factor analysis
Source: J Financ Serv Mark. 2026 Aug 7;31(3):50. doi: 10.1057/s41264-026-00384-5 (PMC13584979; doi:10.1057/s41264-026-00384-5)
Supplement: Supplementary file 5 [file 41264_2026_384_MOESM4_ESM.docx]

Table S3. The eigenvalues of the five dimensions derived statistically by principal component analysis along with the percentage of variance described by the corresponding dimensions.

| eigenvalue | percentage of variance | cumulative percentage of variance |
| --- | --- | --- |
| 1.75346439942743 | 35.0692879885487 | 35.0692879885487 |
| 1.0325144682686 | 20.6502893653721 | 55.7195773539208 |
| 0.815298394206581 | 16.3059678841316 | 72.0255452380524 |
| 0.782939655468227 | 15.6587931093646 | 87.684338347417 |
| 0.615783082629151 | 12.315661652583 | 100 |
