# Supplementary material for: Latent dimensions of financial behaviour derived by principal component and factor analysis
Source: J Financ Serv Mark. 2026 Aug 7;31(3):50. doi: 10.1057/s41264-026-00384-5 (PMC13584979; doi:10.1057/s41264-026-00384-5)
Supplement: Supplementary file 6 [file 41264_2026_384_MOESM5_ESM.docx]

Table S4. The eigenvalues of the underlying factors obtained by principal component analysis along with the percentage of variance described by the corresponding factors.

| eigenvalue | percentage of variance | cumulative percentage of variance |
| --- | --- | --- |
| 2.25135698725379 | 20.4668817023072 | 20.4668817023072 |
| 1.40887527840664 | 12.807957076424 | 33.2748387787312 |
| 1.32884621432952 | 12.0804201302684 | 45.3552589089996 |
| 1.09758352666517 | 9.97803206059243 | 55.333290969592 |
| 0.964247024205462 | 8.76588203823147 | 64.0991730078235 |
| 0.890648008814054 | 8.09680008012776 | 72.1959730879512 |
| 0.804041312631339 | 7.30946647846672 | 79.505439566418 |
| 0.725059341718692 | 6.59144856107902 | 86.096888127497 |
| 0.596531440410564 | 5.42301309464149 | 91.5199012221385 |
| 0.564900262265079 | 5.13545692968253 | 96.655358151821 |
| 0.367910603299689 | 3.34464184817899 | 100 |
