# Supplementary material for: Latent dimensions of financial behaviour derived by principal component and factor analysis
Source: J Financ Serv Mark. 2026 Aug 7;31(3):50. doi: 10.1057/s41264-026-00384-5 (PMC13584979; doi:10.1057/s41264-026-00384-5)
Supplement: Supplementary file 7 [file 41264_2026_384_MOESM6_ESM.docx]

Table S5a. The detailed factor analysis by two factors.

Factor Analysis using method = minres

Call: fa(r = data, nfactors = nfactor, rotate = rotation)

Standardized loadings (pattern matrix) based upon correlation matrix

|  | MR1 | MR2 | h2 | u2 | com |
| --- | --- | --- | --- | --- | --- |
| Inclusion | 0.34 | 0.16 | 0.14 | 0.8561 | 1.4 |
| Literacy | 0.46 | 0.25 | 0.28 | 0.7204 | 1.5 |
| Risk | 0.16 | 0.36 | 0.15 | 0.8482 | 1.4 |
| Trust | 0.32 | 0.38 | 0.25 | 0.7543 | 1.9 |
| Usage | 0.92 | -0.38 | 0.99 | 0.0083 | 1.3 |

|  | MR1 | MR2 |
| --- | --- | --- |
| SS loadings | 1.31 | 0.51 |
| Proportion Var | 0.26 | 0.10 |
| Cumulative Var | 0.26 | 0.36 |
| Proportion Explained | 0.72 | 0.28 |
| Cumulative Proportion | 0.72 | 1.00 |

Mean item complexity = 1.5

Test of the hypothesis that 2 factors are sufficient.

df null model = 10 with the objective function = 0.34 with Chi Square = 158.69

df of the model are 1 and the objective function was 0

The root mean square of the residuals (RMSR) is 0.01

The df corrected root mean square of the residuals is 0.03

The harmonic n.obs is 470 with the empirical chi square 0.61 with prob < 0.44

The total n.obs was 470 with Likelihood Chi Square = 0.47 with prob < 0.49

Tucker Lewis Index of factoring reliability = 1.036

RMSEA index = 0 and the 90 % confidence intervals are 0 0.107

BIC = -5.69

Fit based upon off diagonal values = 1

Measures of factor score adequacy

|  | MR1 | MR2 |
| --- | --- | --- |
| Correlation of (regression) scores with factors | 0.96 | 0.72 |
| Multiple R square of scores with factors | 0.91 | 0.52 |
| Minimum correlation of possible factor scores | 0.83 | 0.05 |
