# Supplementary material for: Latent dimensions of financial behaviour derived by principal component and factor analysis
Source: J Financ Serv Mark. 2026 Aug 7;31(3):50. doi: 10.1057/s41264-026-00384-5 (PMC13584979; doi:10.1057/s41264-026-00384-5)
Supplement: Supplementary file 8 [file 41264_2026_384_MOESM7_ESM.docx]

Table S5b. The detailed factor analysis by three factors.

Factor Analysis using method = minres

Call: fa(r = data, nfactors = nfactor, rotate = rotation)

Standardized loadings (pattern matrix) based upon correlation matrix

|  | MR1 | MR2 | MR3 | h2 | u2 | com |
| --- | --- | --- | --- | --- | --- | --- |
| Inclusion | 0.43 | -0.02 | 0.26 | 0.25 | 0.75 | 1.6 |
| Literacy | 0.60 | 0.06 | -0.21 | 0.41 | 0.59 | 1.3 |
| Risk | 0.25 | 0.37 | -0.02 | 0.20 | 0.80 | 1.8 |
| Trust | 0.40 | 0.24 | 0.08 | 0.22 | 0.78 | 1.7 |
| Usage | 0.59 | -0.36 | -0.01 | 0.48 | 0.52 | 1.7 |

|  | MR1 | MR2 | MR3 |
| --- | --- | --- | --- |
| SS loadings | 1.10 | 0.33 | 0.12 |
| Proportion Var | 0.22 | 0.07 | 0.02 |
| Cumulative Var | 0.22 | 0.29 | 0.31 |
| Proportion Explained | 0.71 | 0.21 | 0.08 |
| Cumulative Proportion | 0.71 | 0.92 | 1.00 |

Mean item complexity = 1.6

Test of the hypothesis that 3 factors are sufficient.

df null model = 10 with the objective function = 0.34 with Chi Square = 158.69

df of the model are -2 and the objective function was 0

The root mean square of the residuals (RMSR) is 0

The df corrected root mean square of the residuals is NA

The harmonic n.obs is 470 with the empirical chi square 0 with prob < NA

The total n.obs was 470 with Likelihood Chi Square = 0 with prob < NA

Tucker Lewis Index of factoring reliability = 1.068

Fit based upon off diagonal values = 1

Measures of factor score adequacy

|  | MR1 | MR2 | MR3 |
| --- | --- | --- | --- |
| Correlation of (regression) scores with factors | 0.80 | 0.58 | 0.38 |
| Multiple R square of scores with factors | 0.64 | 0.33 | 0.15 |
| Minimum correlation of possible factor scores | 0.28 | -0.34 | -0.71 |
